# Absence of Interferon Regulatory Factor 1 Protects Against Atherosclerosis in Apolipoprotein E-Deficient Mice

**DOI:** 10.7150/thno.36862

**Published:** 2019-07-09

**Authors:** Meng Du, Xiaojing Wang, Xiaoxiang Mao, Liu Yang, Kun Huang, Fengxiao Zhang, Yan Wang, Xi Luo, Cheng Wang, Jiangtong Peng, Minglu Liang, Dan Huang, Kai Huang

**Affiliations:** 1 Department of Cardiology, Union Hospital, Tongji Medical College, Huazhong University of Science and Technology, Wuhan, China; 2 Clinic Center of Human Gene Research, Union Hospital, Tongji Medical College, Huazhong University of Science and Technology, Wuhan, China; 3 Department of Geriatrics, Union Hospital, Tongji Medical College, Huazhong University of Science and Technology, Wuhan, China

**Keywords:** IRF1, atherosclerosis, plaque stability, foam cell formation, endotoxemia

## Abstract

Deciphering the molecular and cellular processes involved in foam cell formation is critical to understanding the pathogenesis of atherosclerosis. Interferon regulatory factor 1 (IRF1) was first identified as a transcriptional regulator of type-I interferons (IFNs) and IFN inducible genes. Our study aims to explore the role of IRF1 in atherosclerotic foam cell formation and understand the functional diversity of IRF1 in various cell types contributing to atherosclerosis.

**Methods**: We induced experimental atherosclerosis in ApoE^-/-^IRF1^-/-^ mice and evaluated the effect of IRF1 on disease progression and foam cell formation.

**Results**: IRF1 expression was increased in human and mouse atherosclerotic lesions. IRF1 deficiency inhibited modified lipoprotein uptake and promoted cholesterol efflux, along with altered expression of genes implicated in lipid metabolism. Gene expression analysis identified scavenger receptor (SR)-AI as a regulated target of IRF1, and SR-AI silencing completely abrogated the increased uptake of modified lipoprotein induced by IRF1. Our data also explain a mechanism underlying endotoxemia-complicated atherogenesis as follows: two likely pro-inflammatory agents, oxidized low-density lipoprotein (ox-LDL) and bacterial lipopolysaccharide (LPS), exert cooperative effects on foam cell formation, which is partly attributable to a shift of IRF1-Ubc9 complex to IRF1- myeloid differentiation primary response protein 88 (Myd88) complex and subsequent IRF1 nuclear translocation. Additionally, it seems that improved function of vascular smooth muscle cells (VSMCs) also accounts for the diminished and more stable atherosclerotic plaques observed in ApoE^-/-^IRF1^-/-^ mice.

**Conclusions**: Our findings demonstrate an unanticipated role of IRF1 in the regulation of gene expression implicated in foam cell formation and identify IRF1 activation as a new risk factor in the development, progression and instability of atherosclerotic lesions.

## Introduction

Atherosclerosis is a systemic disease characterized by interactions among multiple kinds of cells which result in local inflammation of the arterial wall 1, 2. As a hallmark of disease development and progression, the roles of macrophage-derived foam cells cannot be ignored. With the release of a variety of cytokines, monocytes recruited to sub-endothelium differentiate into macrophages with increased expression of scavenger receptors (SRs) 3. Uncontrolled uptake of modified lipoproteins and impaired cholesterol efflux result in formation of lipid-laden foam cells. Interferon regulatory factor 1 (IRF1) was first identified as a transcriptional regulator of type-I interferons (IFNs) and IFN inducible genes 4. IRF1^-/-^ mice were susceptible to certain kinds of pathogens, but were particularly found to be resistant to several inflammatory and autoimmune diseases 5-8. Subsequent studies carried out on this transcription factor have revealed a remarkable functional diversity in a variety of biological processes, including immune responses, inflammatory processes, cell growth, apoptosis, and oncogenesis 9-11.

Although a strong positive correlation of IRF1 with oxidized low-density lipoprotein (ox-LDL) in mammalian atherosclerotic lesions has been suggested previously 12, little information is available concerning the effects of IRF1 on foam cell formation and atherosclerosis. Moreover, coexisted low-grade endotoxemia in patients with atherosclerosis may be a new risk factor for the pathogenesis of disease. Despite significant pathological implication of low-grade inflammation in foam cell formation and atherosclerosis 13-15, whether IRF1 is involved in this process also remains elusive. Here, we demonstrate that IRF1 acts as a critical modulator in transforming macrophages into lipid-laden foam cells, and provide novel evidence explaining the link between subclinical endotoxemia and foam cell transformation. In addition, our findings suggest that the mechanisms whereby IRF1 deficiency attenuates and stabilizes atherosclerotic lesions are multifactorial, involving different physiological pathways and a variety of cell types.

## Methods

Details of materials and experimental procedures are available in the Online Data Supplement.

### Human atherosclerotic tissues

Human atherosclerotic lesions were collected from patients undergoing carotid endarterectomy at Wuhan Union Hospital, and internal mammary arteries obtained from patients undergoing coronary artery bypass surgery were used as non-atherosclerotic control arteries. Written informed consent was obtained from all participants according to the declaration of Helsinki. The investigations were approved by the Ethical Committee of Huazhong University of Science and Technology.

### Animals

IRF1 global knockout mice (IRF1^-/-^ on a C57BL/6 background) were kindly provided by Dr Hongliang Li (Wuhan University, Wuhan, China) 16. To obtain the ApoE^-/-^IRF1^-/-^ mice, we generally crossbred the IRF1^-/-^ with ApoE^-/-^ to get ApoE^+/-^IRF1^+/-^ heterozygous mice. ApoE^-/-^IRF1^-/-^ mice and the control ApoE^-/-^ littermates were obtained by inbreeding between ApoE^+/-^IRF1^+/-^ heterozygous mice. At the end of the study, Mice were euthanized via intraperitoneal injection of pentobarbital (150 mg/ kg). All the procedures involving mouse experiments were approved by the Ethics Committee of Union Hospital, Huazhong University of Science and Technology, China, and were conducted in accordance with the National Institutes of Health (NIH) Guide for the Care and Use of Laboratory Animals.

### Histological analysis and quantification of atherosclerotic lesions

Lipid accumulation of thoracoabdominal aorta was determined by *en face* Oil Red O staining. For the microscopic evaluation of the aortic sinus lesions, sections were stained for lipid accumulation with Oil red O, for morphology with hematoxylin and eosin (H&E), for collagen content with Masson's trichrome staining (Masson), for elastic fibers with elastica van Gieson staining (EVG). The relative content of macrophages and smooth muscle cells were detected by immunohistochemistry.

### Generation of recombinant adenovirus

Replication-defective recombinant adenovirus carrying the entire coding sequence of IRF1 (AdIRF1) or shRNA against IRF1 (AdshIRF1) were constructed with Adenovirus Expression Vector Kit (Takara Bio Inc., Kusatsu, Japan). Amplification and purification of recombinant adenovirus was performed according to the manufacturer's instructions (Takara Bio).

### Real-time PCR and western blot

Quantitative polymerase chain reaction (PCR) amplification was performed according to the manufacturer's instructions. The real-time PCR primer sequences are shown in Supplementary Table 1. Cells or tissues were harvested at indicated times and equal amounts of protein were fractionated by SDS polyacrylamide gels, followed by immunoblotting with specific antibodies. Membranes were then incubated with peroxidase-conjugated secondary antibody, and specific bands were detected with a Bio-Rad (Hercules, CA) imaging system.

### Immunoprecipitations and Mass Spectrometry

Immunoprecipitation was performed to determine protein complex formation and coimmunoprecipitated proteins were separated with SDS-PAGE followed by mass spectrometry or Western blot.

### Luciferase assays

Luciferase reporter constructs (SR-AI, CD36, LOX-1, SR-BI, ABCA1 and ABCG1) were co-transfected with an internal control plasmid pRL-TK (Renilla luciferase reporter plasmid, Promega) into HEK293T cells. The luciferase activity was determined with Dual Luciferase Reporter Assay Kit (Promega) according to the manufacturer's instruction.

### Chromatin immunoprecipitation (ChIP) assay

ChIP assay was performed according to the instructions (CHIP assay kit, Millipore) using monoclonal antibodies against IRF1. DNA samples recovered after immunoprecipitation were subjected to PCR.

### Foam cell formation assay and quantification of cholesterol content

Macrophages were incubated with 50 μg/ml ox-LDL for different times, fixed with ethanol, and stained with Oil Red O as described 17. Intracellular cholesterol content was measured as previously described 18.

### Cell culture

Peritoneal macrophages were isolated from C57BL/6 mice 19. Primary SMCs were obtained from 6 to 8-week-old C57 male mouse aortas using collagenase-elastase digestion 20. HEK293T cells (CRL-11268), THP-1 cells (TIB-202) and T/G HA-VSMC (CRL-1999) were obtained from ATCC and cultured according to the manufacturer's instructions. All of the cell lines were free of mycoplasma contamination (tested by the vendors using the MycoAlert kit from Lonza). No cell lines used in this study are found in the database of commonly misidentified cell lines (ICLAC and NCBI Biosample) based on short tandem repeats (STR) profiling performed by vendors.

### Statistical analysis

GraphPad Prism software (GraphPad Software Inc., La Jolla, CA) was used for statistical analyses. Band intensity in western blot images was quantified with Image J Software. Values are expressed as means ± SEM of at least three independent experiments. Student's t test was used to assess the statistical significance of the differences between two groups. Benjamini-Hochberg corrections for multiple variables were used in transcriptional studies. For multiple groups, significance was evaluated by one-way ANOVA with Bonferroni test (homogeneity of variance) or Tamhanes's T2 test (heterogeneity of variance). *P* < 0.05 was considered statistically significant. Randomization and blinding strategy was used whenever possible. Animal cohort sizes were determined on the basis of similar previous studies.

## Results

### Expression of IRF1 is increased in human and mouse atherosclerotic lesions

**Figure 1:**
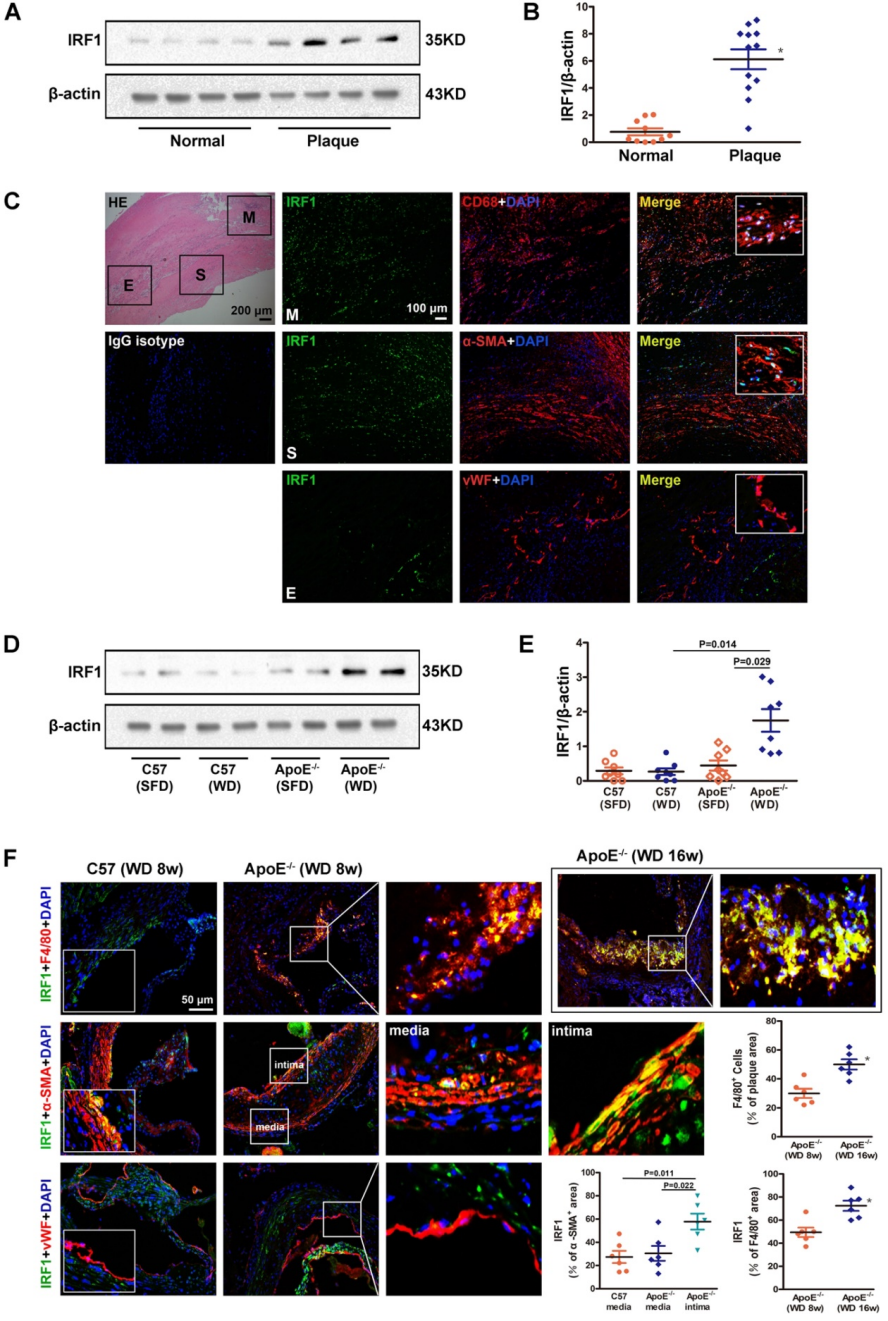
** IRF1 expression in atherosclerotic lesions from human and mice. A,** Western blots of IRF1 in human carotid atherosclerotic lesions (n=12) and internal mammary arteries (n=10). **B,** Quantification of band density in panel** A**. **C,** Immunofluorescence assay of IRF1 in human carotid atherosclerotic lesion. Sections were co-stained for IRF1 (green) and cell specific markers (red; CD68 for macrophage, α-SMA for smooth muscle cell, von Willebrand Factor for endothelial cell). 4',6-diamidino-2-phenylindole (DAPI) was used for nucleus staining (blue). M, Macrophage-rich areas; S, VSMC-rich areas; E, Endothelium. Scale bar = 100 μm. **D,** Western blots of IRF1 in atherosclerotic mice fed with western diet or chow diet (n=8 for each group). **E,** Quantification of band density in panel** D**.** F,** Immunofluorescence staining and quantification **(*lower right*)** of IRF1 in atherosclerotic lesions of aortic sinus from ApoE^-/-^ mice (F4/80 for macrophage, α-SMA for smooth muscle cell, von Willebrand Factor for endothelial cell). Scale bar = 50 μm. Data are expressed as mean ± SEM. One-way ANOVA with Tamhanes's T2 test was used to produce the *P* values given in panel **E**. One-way ANOVA with Bonferroni test was used to produce the *P* values given in panel **F**. * *P* < 0.05 vs. control group.

To clarify the role of IRF1 in atherogenesis, we firstly examined the level of IRF1 in human carotid atherosclerotic lesions and internal mammary arteries (healthy vessels). As shown in Figure 1A and B, IRF1 protein level was markedly increased in plaques versus control vessels. As double immunofluorescent staining demonstrated, IRF1, in atherosclerotic lesions, was expressed predominantly in smooth muscle cells (SMCs) and macrophages that exhibited positive staining for α-SMA or CD68 respectively (Figure 1C). IRF1 was barely expressed in endothelial cells lining the neovascular (Figure 1C). Further we examined the expression of IRF1 in atherosclerosis- prone ApoE^-/-^ mice and normal C57BL/6 mice. As expected, IRF1 protein level in aorta of ApoE^-/-^ mice was significantly increased after 8 weeks of western diet feeding compared to that on standard chow diet or C57BL/6 mice (Figure 1D and E). Consistently, evident immunoreactivity for IRF1 was observed in smooth muscle cells and macrophages in lesions of aortic sinus from ApoE^-/-^ mice (Figure 1F). These data suggest a potential role of IRF1 in the development of atherosclerosis, as macrophages and SMCs are major cell types contributing to atherogenesis. To determine whether IRF1 responds to the progression of atherosclerosis, we detected its levels in macrophages from early- and late-stage lesions respectively. The immunostaining in the lesions of aortic sinus revealed increased IRF1 expression in macrophages from ApoE^-/-^ mice fed western diet for 16 weeks than in mice fed for 8 weeks, IRF1 stained areas were quantified as a percentage of F4/80^+^ areas (Figure 1F). Moreover, we also detected IRF1 expression in smooth muscle cells from tunica media and proliferated intima of atherosclerotic lesions. IRF1 expression of SMCs in the media of diseased vessel was almost unchanged compared with normal vessel. However, the level of IRF1 in proliferated intima SMCs was markedly increased compared with that from media or normal vessels (Figure 1F).

### IRF1 deficiency restricts the development of atherosclerosis

**Figure 2:**
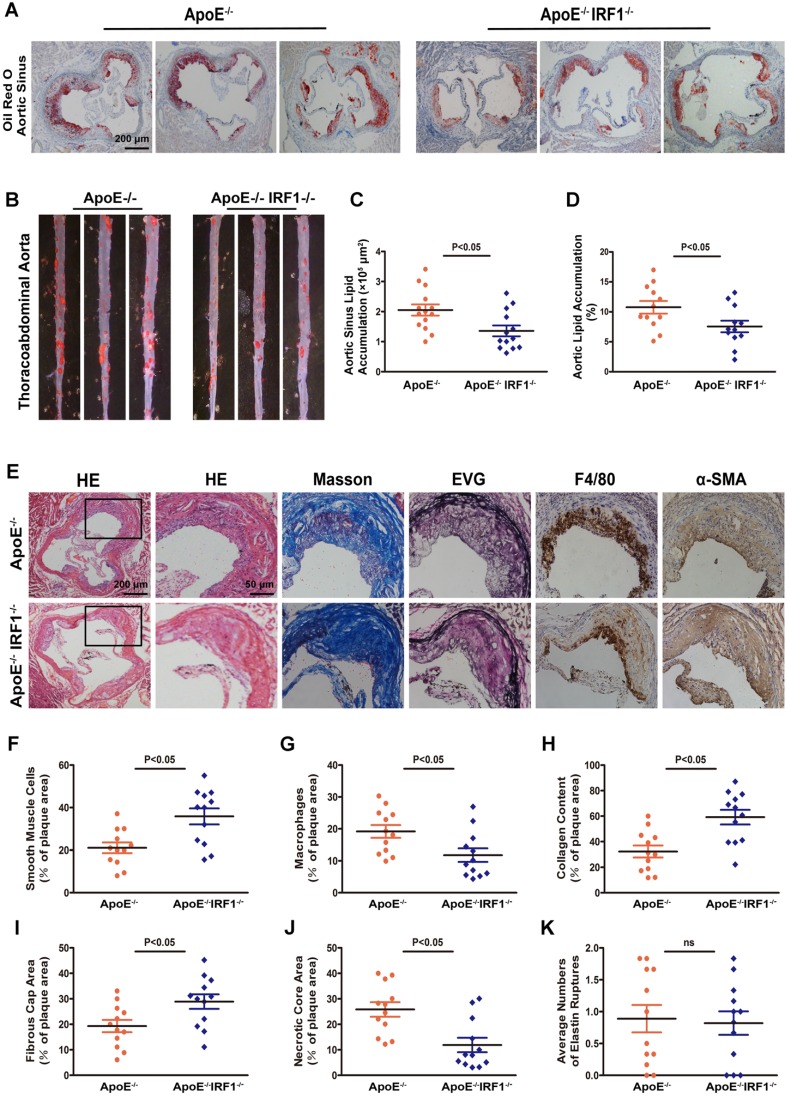
** IRF1 deficiency restricts the development of atherosclerosis.** ApoE^-/-^ and ApoE^-/-^IRF1^-/-^ mice were fed a western diet for 16 weeks. **A and C,** Images and quantification of Oil Red O staining in lesions of aortic sinus. Scale bar = 200 μm. **B and D,** Oil Red O staining of thoracoabdominal aorta, and lipid accumulation was quantified as percentage of total surface area of aorta. **E,** Representative images of aortic sinus for Hematoxylin and Eosin (HE), Masson's trichrome (Masson), Elastica van Gieson (EVG), smooth muscle cells (α-SMA) and macrophages (F4/80). Scale bar = 50 μm. **F-K,** Quantification of collagen content, fibrous cap area, necrotic core area, ruptures of elastic fibers, macrophage content and smooth muscle cell content. Data are expressed as mean ± SEM (n=12 per group). Student's t test was used to produce the *P* values given in figure.

Atherosclerotic lesion formation was assessed after 16-week of western diet feeding. There was no difference in body weight, serum cholesterol level, and serum triglyceride level between ApoE^-/-^ and ApoE^-/-^IRF1^-/-^ mice (Supplemental Table 2). Analysis of Oil Red O staining in the aortic sinus showed a significant decrease (33.8%) in lipid accumulation of ApoE^-/-^IRF1^-/-^ mice compared with control ApoE^-/-^ mice (Figure 2A and C), and there was a consistent 29.8% decrease in lipid accumulation at the thoracoabdominal aorta in ApoE^-/-^IRF1^-/-^ mice (Figure 2B and D). These results suggest that IRF1 may play an important role in the aggravation of atherosclerosis.

### Effects of IRF1 deficiency on lesional morphology of atherosclerotic plaque

The complexity of atherosclerotic lesions was assessed after 16-week of western diet feeding. Plaque composition, with regard to macrophage (F4/80- positive) content, smooth muscle cell (α-SMC- positive) and collagen (Masson's Trichrome stain) content, fibrous cap area and necrotic croe area, and the destruction of the elastic laminae (Verhoeff's Van Gieson stain), was significantly altered by IRF1 deficiency (Figure 2E). Compared with ApoE^-/-^ mice, plaques of ApoE^-/-^IRF1^-/-^ mice contained a relatively integral VSMC-rich fibrous cap with abundant collagen and matrix overlying smaller necrotic cores. In addition, macrophage content was considerably decreased within lesions from ApoE-/-IRF1-/- mice (Figure 2F-K). These results suggest that IRF1 deficiency induces a highly characteristic architecture of more-stable plaques.

### IRF1 deficiency promotes proliferation and inhibits apoptosis of VSMCs

As an important component of atherosclerotic lesions, VSMCs exert various effects in the disease. Moderate proliferation of VSMCs favors plaque stability in advanced lesions, and increased apoptosis rate may lead to plaque rupture 21. We next investigated whether IRF1 deficiency induces features of plaque stability by directly regulating VSMC function. The immunostaining in the lesions of aortic sinus revealed markedly increased Ki67 positive cells in areas rich in smooth muscle cells of ApoE^-/-^IRF1^-/-^ mice compared with ApoE^-/-^ mice (Supplemental Figure 1A and C). Concomitantly, a moderate decrease in apoptotic smooth muscle cells (Tunel^+^ α-SMA^+^) was also observed in ApoE^-/-^IRF1^-/-^ mice (Supplemental Figure 1B and D). The effects of IRF1 on VSMC proliferation, migration and apoptosis were confirmed in primary mice VSMCs cultured *in vitro*. Adenovirus-mediated gene manipulation was employed to enforce (AdIRF1 and control AdGFP) or silence (AdshIRF1 and control AdshRNA) the expression of IRF1. Equal number of cells were seeded into 12-well plates and cultured for 72 hours in medium containing 10% fetal bovine serum (FBS). As expected, knockdown of IRF1 significantly increased the cell number at 48 and 72 hours, whereas overexpression of IRF1 had the opposite effects (Supplemental Figure 1E and F). In addition, as assessed using a transwell assay, the migration of VSMCs was promoted by IRF1 silencing and inhibited by IRF1 overexpression, either in resting condition or induced by 10% FBS (Supplemental Figure 1G-I). Next, the effects of IRF1 on VSMC apoptosis were analyzed. As shown in Supplemental Figure 1J, and quantified in Supplemental Figure 1K-L, the incubation with ox-LDL caused a dramatic increase of Tunel positive VSMCs, and these pro-apoptotic effects were partly reversed by silencing of IRF1; in contrast, enforcing IRF1 expression further promoted the apoptosis of VSMCs induced by ox-LDL. Collectively, these data indicated that IRF1 deficiency not only promoted the proliferation and migration of VSMCs, but also inhibited VSMC apoptosis, thus inducing a more stable plaque phenotype.

### IRF1 contributes to foam cell formation

**Figure 3:**
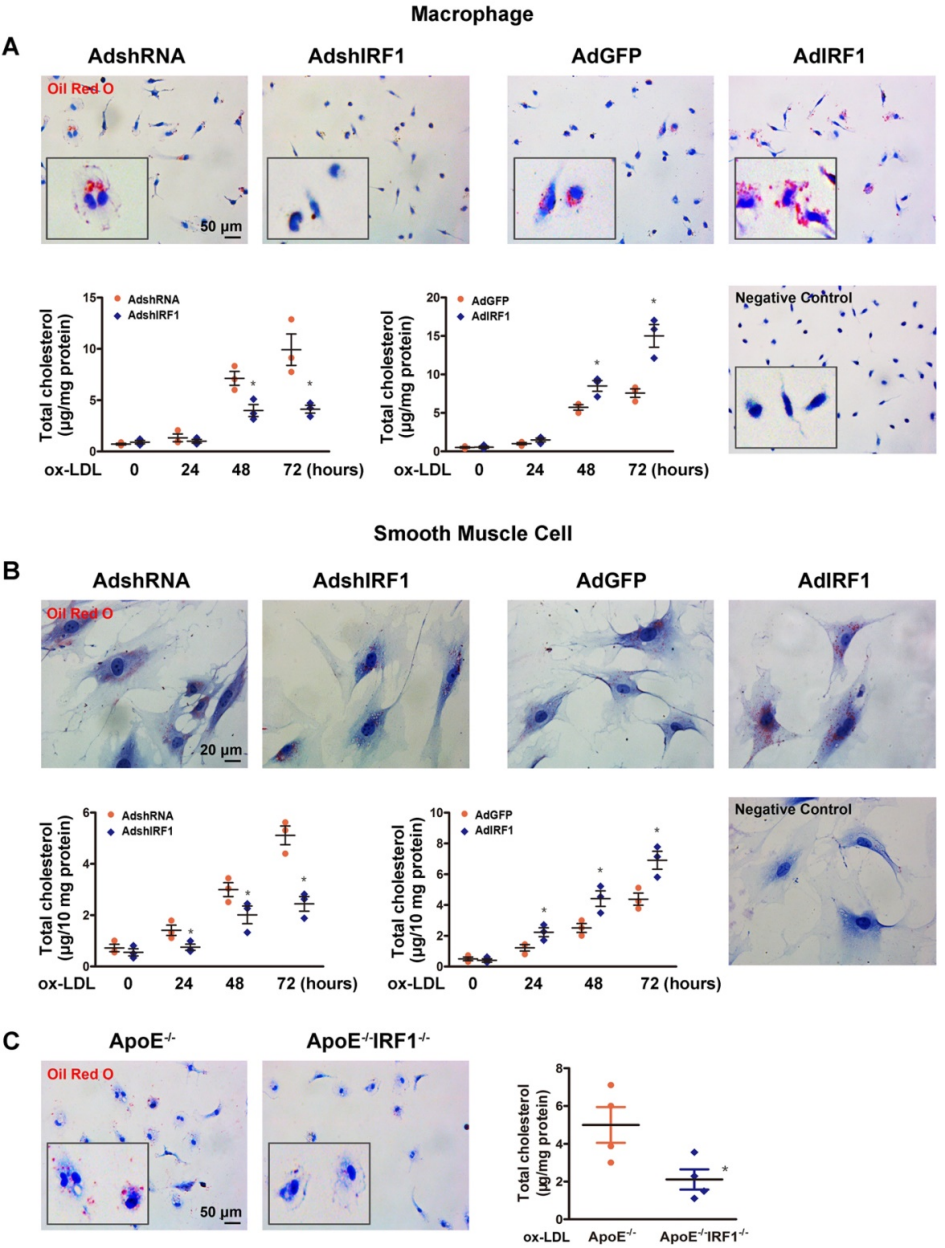
** IRF1 contributes to foam cell formation. A and B,** IRF1 expression was silenced (AdshIRF1 and control AdshRNA) or enforced (AdIRF1 and control AdGFP) in mice peritoneal macrophages and primary smooth muscle cells cultured *in vitro*. Oil Red O staining images and determination of cholesterol content in macrophages **(A)** and smooth muscle cells **(B)** incubated with oxidized low-density lipoprotein (ox-LDL, 50 μg/mL) for different times. Images of negative control stained for nuclei alone were shown on ***lower right***. Scale bar = 50 μm in panel **A**. Scale bar = 20 μm in panel **B**. Data represent the mean ± SEM of three independent experiments. * *P* < 0.05 vs. AdshRNA group or AdGFP group. **C,** Representative images of Oil Red O staining and determination of cholesterol content of peritoneal macrophages derived from ApoE^-/-^IRF1^-/-^ and ApoE^-/-^mice. Scale bar = 50 μm. Data are expressed as mean ± SEM (n=4 per group). * *P* < 0.05 vs. ApoE^-/-^ mice.

The studies described above showed that lesions in ApoE^-/-^IRF1^-/-^ mice were less lipid and macrophage rich. To determine whether IRF1 affects foam cell formation, Oil Red O staining and quantification of cholesterol content were performed in primary peritoneal macrophages isolated from mice. As shown in Figure 3A, incubation with ox-LDL increased macrophage lipid accumulation in a time-dependent manner. Macrophage silenced with AdshIRF1 showed a significant decrease in both Oil Red O staining and cholesterol content when compared to that treated with AdshRNA. In contrast, enforcing IRF1 expression by AdIRF1 dramatically increased the capacity of macrophages to form foam cells. It has been confirmed that the presence of SMC-derived foam cells in atherosclerotic lesion also plays a critical role in early stage and progression of atherosclerosis 22. We also demonstrated that silencing or enforcing IRF1 expression in primary cultured smooth muscle cells had similar effects on foam cell formation as in macrophages (Figure 3B).To further confirm these results, we assessed the lipid accumulation in peritoneal macrophages derived from ApoE^-/-^ or ApoE^-/-^ IRF1^-/-^ mice on exposure to ox-LDL. Quantitatively, the total cholesterol in macrophages derived from ApoE^-/-^IRF1^-/-^ mice was 57.7% less than in ApoE^-/-^ macrophages (Figure 3C). Moreover, a similar dependence of lipid accumulation on IRF1 was observed in human THP-1 macrophages (Supplemental Figure 2A and B) and a human VSMC line (T/G HA-VSMC) derived from normal aortic VSMCs (Supplemental Figure 2C and D). These results indicated that IRF1 deficiency inhibits foam cell formation of macrophages and smooth muscle cells.

### IRF1 deficiency inhibits the uptake of modified lipoproteins and promotes cholesterol efflux

Uncontrolled uptake of modified lipoproteins and impaired cholesterol efflux lead to foam cell formation 23. Our results revealed that the uptake of fluorescently labeled ox-LDL (Dil-ox-LDL), as examined using flow cytometry analysis, was significantly decreased in IRF1-silenced macrophages and smooth muscle cells, whereas an opposite effect was observed in IRF1 over-expressed cells (Supplemental Figure 3A-D). Next, the cholesterol efflux assay was performed, as demonstrated in Supplemental Figure 3E and F, IRF1 knockdown markedly increased ApoAI-dependent cholesterol efflux in lipid laden macrophages and smooth muscle cells, and IRF1 overexpression exerted an opposite effect. In contrast, the cholesterol efflux to HDL was unaffected by IRF1. To further confirm these results, we injected DiI-ox-LDL into ApoE^-/-^ and ApoE^-/-^ IRF1^-/-^ mice. There was a 55.9% decrease in DiI labeling in the aortic sinus of ApoE^-/-^IRF1^-/-^ mice than in ApoE^-/-^ mice (Supplemental Figure 3G and H). Together these results suggest that IRF1 deficiency inhibits foam cell formation through decreased uptake of modified lipoproteins and increased cholesterol efflux.

### IRF1 regulates gene expression related to foam cell formation

**Figure 4:**
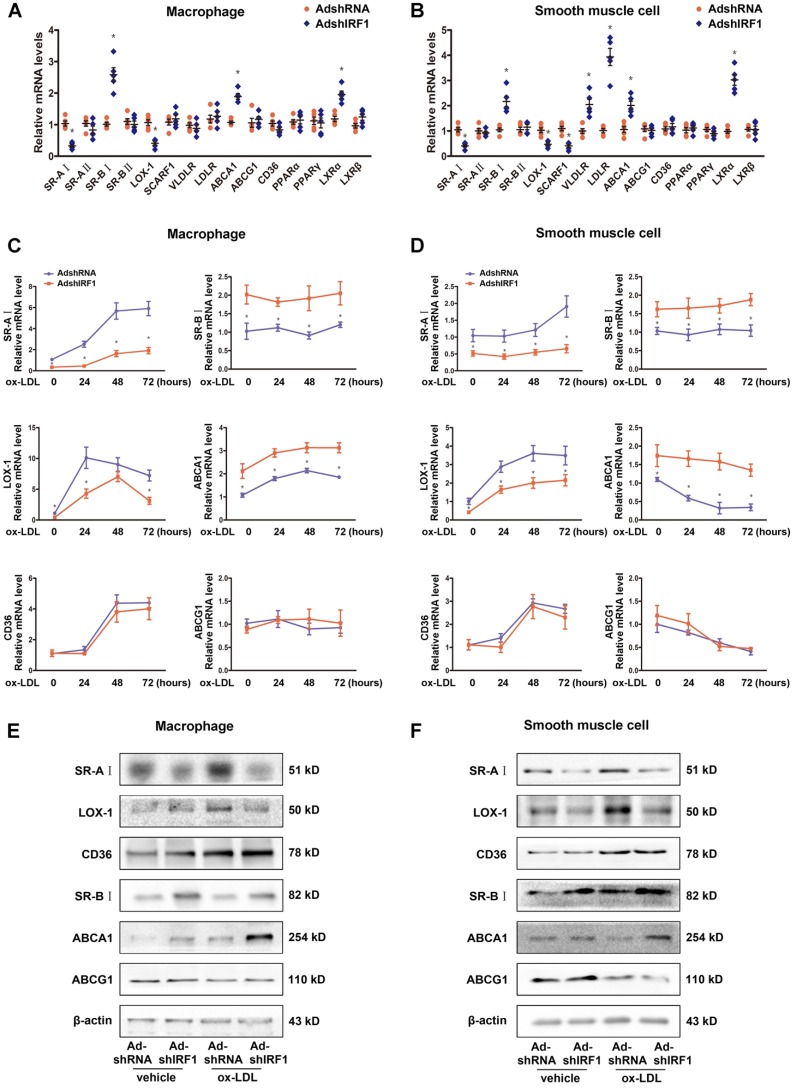
** IRF1 regulates gene expression related to foam cell formation. A and B,** mRNA levels of genes related to foam cell foamation in mice peritoneal macrophages **(A)** and primary smooth muscle cells **(B)** silenced with AdshIRF1 or AdshRNA. **C and D,** Mice peritoneal macrophages **(C)** and primary smooth muscle cells** (D)** silenced with AdshIRF1 or AdshRNA were incubated with ox-LDL (50 μg/mL), and the relative mRNA levels of SR-AI, LOX-1, CD36, SR-BI, ABCA1 and ABCG1 were examined at different time points. Data represent the mean ± SEM of three to five independent experiments. Benjamini-Hochberg corrections for multiple variables were used in transcriptional studies. * *P* < 0.05 vs. AdshRNA group. **E and F,** Representative immunoblot for SR-AI, LOX-1, CD36, SR-BI, ABCA1 and ABCG1 in lipid-laden macrophages **(E)** and smooth muscle cells **(F)** silenced with AdshIRF1 or AdshRNA.

To gain insights into potential mechanisms by which IRF1 knockdown blunted foam cell formation, we assessed the expression of genes implicated in lipid metabolism. These include receptors involved in ox-LDL uptake, receptors involved in phagocytosis, transporters involved in cholesterol efflux and transcription factors known to regulate these receptors and transporters. The expression of SR-AI and LOX-1, which are the principal receptors responsible for the uptake of modified lipoproteins, were significantly decreased in IRF1-silenced macrophages, whereas the levels of transporters responsible for cholesterol efflux, SR-BI and ABCA1, were increased (Figure 4A). Importantly, primary smooth muscle cells showed a similar gene expression tendency (Figure 4B). The difference is that, silencing IRF1 also inhibited the expression of receptor SCARF1 and promoted the expression of LDLR (low-density lipoprotein receptor) and VLDLR (very low-density lipoprotein receptor) in smooth muscle cells (Figure 4B). In contrast, no appreciable changes of genes related to macrophage polarization were observed. Also, genes involved in phagocytosis and efferocytosis were almost unaffected by IRF1 knockdown except for C1qa (Supplemental Figure 4A). We further examined the expression of these related genes in ox-LDL loaded macrophages and smooth muscle cells. As shown in Figure 4C and D, ox-LDL stimulation induced an increase in SR-AI and LOX-1 levels of macrophages and smooth muscle cells, whereas silencing IRF1 blunted this impact. For the transports responsible for cholesterol efflux, the expression of ABCA1 was increased in macrophages and decreased in smooth muscle cells during foam cell formation, and the level of SR-BI remained unchanged. However, both under basal conditions and upon ox-LDL stimulation, the abundance of two transports were higher in IRF1-silenced cells. In contrast, the expression of CD36 and ABCG1 were unaffected by IRF1 knockdown. Determination of protein levels of these genes further confirmed our results (Figure 4E and F). Consistently, a decrease in SR-AI level and an increase in ABCA1 level were also observed in atherosclerotic lesions of ApoE^-/-^IRF1^-/-^ mice compared to control ApoE^-/-^ mice (Supplemental Figure 4B and C).

### IRF1 facilitates SR-AI expression at transcriptional level

**Figure 5:**
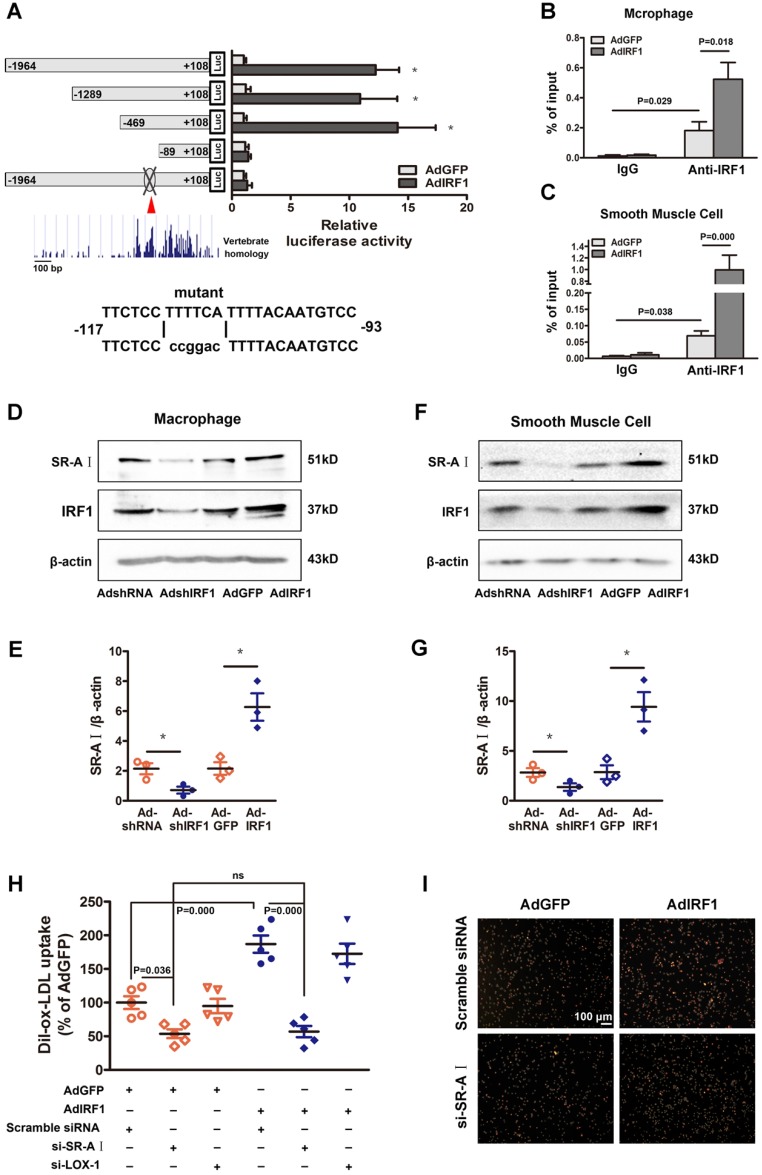
** IRF1 facilitates SR-AI expression at transcriptional level. A,** Luciferase reporter constructs containing murine SR-AI promoter truncations or its mutants were transfected along with pRL-TK (internal control plasmid) into HEK293T cells, followed by infection with AdIRF1 or AdGFP. The relative luciferase activity is quantified as a percent of value determined in AdGFP group. **B and C,** Chromatin immunoprecipitation (ChIP) assay revealed the affinity of IRF1 on SR-AI promoter in mice peritoneal macrophages **(B)** and smooth muscle cells **(C)**. **D,** Representative immunoblot for SR-AI and IRF1 in mice peritoneal macrophages infected with AdshIRF1 or AdIRF1. **E,** Quantification of band density in** D**. **F,** Representative immunoblot for SR-AI and IRF1 in mice primary smooth muscle cells infected with AdshIRF1 or AdIRF1. **G,** Quantification of band density in** F. H and I,** The uptake of fluorescently labeled ox-LDL (Dil-ox-LDL) in IRF1 over-expressed macrophages silenced with si-SR-AI or si-LOX-1, as determined by flow cytometry **(H)** and displayed in fluorescence images **(I)**. Scale bar = 100 μm. Data represent the mean ± SEM of three to five independent experiments. One-way ANOVA with Bonferroni test was used to produce the *P* values given in figure. * *P* < 0.05. ns, no significance.

To determine whether IRF1 could regulate the transcription of genes involved in foam cell formation, the 2000 bp promoter-luciferase reporter constructs were established and transfected into cultured HEK293T cells. IRF1 overexpression significantly increased the luciferase activity of SR-AI promoter constructs and had no effects on other genes including LOX-1, CD36, SR-BI, ABCA1 and ABCG1 (Supplemental Figure 4D). Then the constructs containing SR-AI promoter truncations were established and transfected along with AdGFP or AdIRF1. IRF1-dependent SR-AI promoter activation was maintained upon 5′ deletion to -469bp. Further nucleotide deletion to -89bp completely abrogated the luciferase activity induction by IRF1 (Figure 5A). We further performed *in silico* analysis of this sequence (-469bp to -89bp), which was relatively conserved across vertebrate species, and found an IRF1 binding site, also known as IFN- stimulated response element (ISRE). Mutation of this site completely abrogated the effect of IRF1 on SR-AI promoter activation (Figure 5A). Chromatin immunoprecipitation (ChIP) assay was further performed and confirmed that IRF1 showed high enrichment in the ISRE of the SR-AI promoter in macrophages and smooth muscle cells (Figure 5B and C). Consistently, enforcing IRF1 expression elicited a robust induction of SR-AI protein, and silencing IRF1 exerted an opposite effect (Figure 5D-G). Importantly, we also identified the IRF1 binding site within regions of high homology in the promoter of human SR-AI gene by luciferase reporter gene saasy (Supplemental Figure 4E), and verified the occupancy of IRF1 on this specific binding site in human THP-1 cells with ChIP assay (Supplemental Figure 4F). In addition, the protein level of SR-AI in THP-1 cells was consistently regulated by IRF1 as in cells derived from mice (Supplemental Figure 4G and H). Together, these results illustrated that IRF1 could directly bind to ISRE within the promoter of SR-AI gene to regulate its expression.

### IRF1 promotes lipoprotein uptake through upregulating SR-AI

Since the expression of SR-AI and LOX-1 were both regulated by IRF1 (Figure 4A and B), we next determined which receptor could be predominant in IRF1-induced uptake of modified lipoprotein. As shown in Figure 5H and I, enforcing IRF1 expression significantly increased the uptake of Dil-ox-LDL in macrophages, and the effect was abrogated specifically by SR-AI silencing, not LOX-1 silencing. It indicates that the uptake of modified lipoprotein induced by IRF1 is dependent on the upregulation of SR-AI rather than LOX-1. It is probably because that LOX-1 is mainly expressed and acts as the major ox-LDL receptor in endothelial cells 24, whereas its roles in macrophages are relatively limited. In addition, we also demonstrated that silencing of ABCA1 completely reversed the increased cholesterol efflux to ApoAI induced by IRF1 knockdown, whereas silencing of SR-BI had no effects (Supplemental Figure 4I).

### Low-dose LPS promotes foam cell formation by facilitating Myd88-IRF1 interaction and IRF1 nuclear translocation

**Figure 6:**
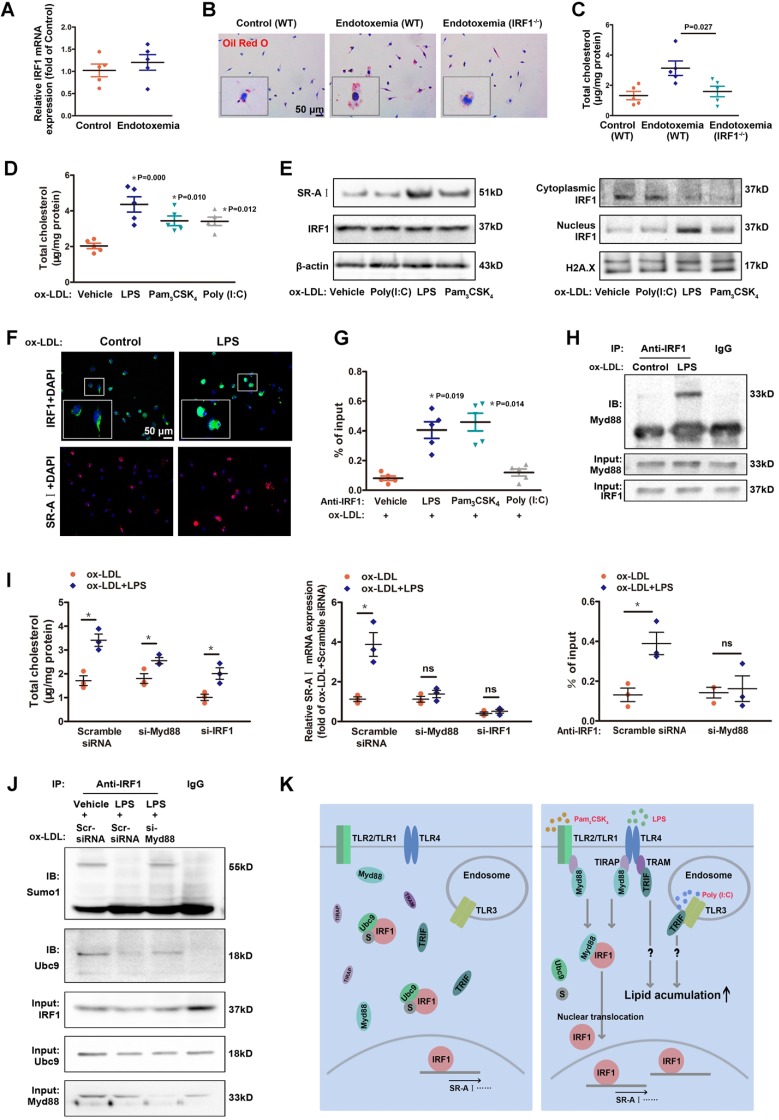
** Low-dose LPS promotes foam cell formation by facilitate Myd88-IRF1 interaction and IRF1 nuclear translocation. A,** The relative mRNA levels of IRF1 in peritoneal macrophages derived from endotoxemia mice and control mice. **B,** Representative images of Oil Red O staining in ox-LDL (50 μg/mL) incubated peritoneal macrophages derived from wild type (WT) and IRF1^-/-^ mice with or without endotoxemia. Scale bar = 50 μm.** C,** Total cholesterol content was measured in ox-LDL incubated peritoneal macrophages derived from WT and IRF1^-/-^ mice with or without endotoxemia. **D,** Total cholesterol content was measured in ox-LDL incubated peritoneal macrophages treated with TLR agonists, that is, LPS (TLR4, 50 pg/mL), Pam_3_CSK_4_ (TLR2/1, 300 ng/mL) and Poly (I:C) (TLR3, 25 μg/mL). **E,** Effects of TLR agonists on IRF1 nuclear translocation and SR-AI expression in macrophages incubated with ox-LDL, as determined by Western blot. **F,** Effects of LPS on IRF1 nuclear translocation (green) and SR-AI expression (red) in macrophages incubated with ox-LDL, as determined by immunofluorescence assay. Scale bar = 50 μm.** G,** Effects of TLR agonists on the interaction of IRF1 with SR-AI promoter in macrophages incubated with ox-LDL, as determined by ChIP assay. **H,** Immunoprecipitation with the control IgG or an anti-IRF1 antibody from ox-LDL incubated macrophages with or without LPS challenge, followed by immunoblot analysis with antibody to Myd88.** I,** Effects of LPS on cholesterol content **(*left panel*)**, SR-AI expression **(*middle panel*)** and the interaction of IRF1 with SR-AI promoter **(*right panel*)** in ox-LDL incubated macrophages silenced with si-Myd88 or si-IRF1. Data represent the mean ± SEM of three to five independent experiments. One-way ANOVA with Bonferroni test was used to produce the *P* values given in figure. * *P* < 0.05. ns, no significance.** J,** Ox-LDL incubated macrophages were silenced with scramble siRNA or si-Myd88 followed by LPS challenge (50 pg/mL), then the lysates were subjected to immunoprecipitation with the control IgG or an anti-IRF1 antibody and analyzed by Western blot with antibodies against Ubc9 and Sumo1. **K,** Schematic diagram of the molecular mechanisms of IRF1 nuclear translocation in foam cells challenged with TLR agonists.

Coexisted low-grade endotoxemia in patients with atherosclerosis may be a new risk factor for the pathogenesis of disease. Both internal and external risk factors including chronic infection, obesity and ageing often lead to mucosal leakages and subclinical levels of circulating bacteria endotoxin liposaccharide (LPS) 14, 25. Despite significant pathological implication of low-grade inflammation in atherosclerosis 13, 14, the fundamental mechanisms are not well understood. In our study, there was no significant difference in IRF1 level of peritoneal macrophages derived from endotoxemia mice and control mice (Figure 6A). However, macrophages from endotoxemia mice had an increased susceptibility to form foam cells (Figure 6B and C). Interestingly, theses effects were nearly ablated in macrophages from IRF1^-/-^ mice (Figure 6B and C), indicating that IRF1 might play an important role in mediating the pro-atherosclerotic effects of endotoxemia. Consistently, treatment with low-dose LPS (50 pg/mL) induced an increase in cholesterol content in cultured macrophages incubated with ox-LDL. In addition, Pam_3_CSK_4_ (TLR1/2 agonist) and Poly (I:C), which is a synthetic double-stranded RNA (TLR3 agonist), also exerted a similar effect on macrophage lipid accumulation (Figure 6D). Further studies were conducted to clarify the mechanisms. Surprisingly, although total IRF1 protein level was unaffected, LPS or Pam_3_CSK_4_ led to a substantial increase in IRF1 nuclear accumulation, along with increased expression of SR-AI, as demonstrated in Western blot analysis (Figure 6E) and immunofluorescence assay (Figure 6F). This is consistent with the observation that IRF1 showed higher enrichment in the specific binding site indentified previously within SR-AI promoter upon LPS or Pam_3_CSK_4_ treatment (Figure 6G). Moreover, we noticed that the effect of LPS on IRF1 nuclear translocation was rapid, as early as at 15 minutes and increased within 1 hour (Supplemental Figure 5A). In contrast, Poly (I:C) had no effects on IRF1 nuclear translocation and SR-AI transcriptional activation (Figure 6E and G). Therefore, rather than alters the protein level of IRF1, LPS and Pam_3_CSK_4_ likely regulates IRF1 subcellular localization in macrophages. However, in basal conditions without ox-LDL challenge, neither LPS nor Pam_3_CSK_4_ failed to induce IRF1 nuclear translocation and recruitment to SR-AI promoter (Supplemental Figure 5B and C). To clarify the mechanisms, we examined the expression and distribution of IRF1 in macrophages with or without ox-LDL treatment. As shown in Supplemental Figure 5D-F, IRF1 was mainly located in the nucleus in basal conditions; however, ox-LDL challenge increased its expression and induced an abundance of IRF1 in both nucleus and cytoplasm. This may account for the necessity of ox-LDL in LPS-induced IRF1 translocation. To confirm our speculation, IFN-γ, also known as typeⅡ immune interferon, which has been proved effective for IRF1 induction 26, was employed in the following study. As expected, IFN-γ also induced a increased in IRF1 protein enriched in cytoplasm and nucleus (Supplemental Figure 5D-F), and treatment with LPS or Pam3CSK4 combined with IFN-γ clearly led to IRF1 translocation from cytoplasm into nucleus followed by transcriptional activation of SR-AI (Supplemental Figure 5G and H). To gain insight into the molecular mechanisms underlying LPS-induced nuclear translocation of IRF1, immunoprecipitation assay in ox-LDL loaded macrophages was employed and proteins interacted with IRF1 were indentified by mass spectrometry. There were 38 proteins in the IRF1 complex in control group without LPS treatment; although 42 interacting proteins were identified in macrophages challenged with LPS, only 32 proteins overlapped with the control group. The results suggested that LPS stimulation led to a dramatic shift of IRF1-binding protein profile (Supplemental Figure 6A). Considering that LPS-induced IRF1 translocation could be associated with gain or loss of interaction between IRF1 and certain proteins, we paid attention to myeloid differentiation primary response gene 88 (MyD88) on the basis of its potential functional relevance in LPS signalling pathway and relatively high reliability score in mass spectrometry analysis. Co-immunoprecipitation experiments confirmed the interaction of IRF1 and Myd88 in macrophages activated by LPS (Figure 6H). Noteworthy, in macrophages incubated with ox-LDL, knockdown of either Myd88 or IRF1 completely reversed the increase in cholesterol content and SR-AI mRNA level induced by Pam_3_CSK_4_ (Supplemental Figure 6B). Consistently, the recruitment of IRF1 to SR-AI promoter was nearly abrogated when Myd88 was knocked down (Supplemental Figure 6B). However, for LPS challenge, silencing of Myd88 or IRF1 only exerted partial inhibitory effects on lipid accumulation in macrophages, although the transcriptional activation of SR-AI was completely inhibited (Figure 6I). This suggests that, besides LPS-Myd88-IRF1-SR- AI pathway, there may be other mechanisms involved in LPS-mediated lipid accumulation and foam cell formation. In addition, the increased cholesterol content in macrophages induced by Poly (I:C) was not affected by Myd88 or IRF1 knockdown (Supplemental Figure 6C), consistent with the fact that Poly (I:C) failed to induce IRF1 nuclear translocation and SR-AI activation (Figure 6E and G). It indicates that Poly (I:C)-mediated aggravation in foam cell formation is independent of IRF1, and the mechanisms need further study.

### SUMOylation of IRF1 by Ubc9 inhibits IRF1 nuclear translocation

Although the association between Myd88 and IRF1 clearly led to IRF1 translocation from cytoplasm to nucleus, Myd88 itself remained in cytoplasm and had not been recruited to SR-AI promoter in response to LPS stimulation (Supplemental Figure 6D and E). It means that Myd88 did not simply act as a carrier of IRF1 for transportation. In contrast to gain of interaction between IRF1 and Myd88, Ubc9, a unique small ubiquitin-like modifier (SUMO) E2-conjugating enzyme responsible for substrate recognition 27, was disassociated from IRF1 with LPS challenge (Supplemental Figure 6A). Co-immunoprecipitation combined with immunoblotting analysis demonstrated that, Ubc9 associated with IRF1 and facilitated its SUMOylation in macrophages loaded with ox-LDL, and that stimulation with LPS definitely inhibited the endogenous Ubc9-IRF1 interaction accompanied with restrained IRF1 SUMOylation (Figure 6J). However, these effects of LPS were abolished when Myd88 was knocked down (Figure 6J).The results indicated that, LPS-activated Myd88 competitively bound to IRF1 and disrupted its interaction with Ubc9, thereby restraining IRF1 SUMOylation. We next determined whether knockdown of Ubc9 could effectively activate IRF1. Unexpectedly, although Ubc9 silencing significantly inhibited IRF1 SUMOylation, it failed to promote IRF1-Myd88 interaction without LPS challenge (Supplemental Figure 6F), and the translocation of IRF1 and SR-AI level were unaffected (Supplemental Figure 6G and H). Moreover, overexpression of Ubc9 could partly reverse the inhibitory effects of LPS on IRF1 SUMOylation, and attenuate the binding of IRF1 to Myd88, thus inhibiting IRF1 translocation and SR-AI activation (Supplemental Figure 6I-K). These results suggest that, the LPS-induced Myd88-IRF1 interaction, as well as the disassociation of Ubc9 from IRF1 accompanied by diminished IRF1 SUMOylation, were both indispensable for the nuclear translocation and activation of IRF1. Further, a glutathione-S-transferase (GST) precipitation assay using recombinant proteins demonstrated direct interaction between IRF1 and Ubc9 (Supplemental Figure 6L). However, we failed to detect the interaction of IRF1 with Myd88 in GST pull-down assay (Supplemental Figure 6M). This might be because LPS signalling activation led to conformational change of Myd88, or formation of some certain protein complex containing other adaptor proteins, which definitely mediated the interaction of Myd88 to IRF1. Nevertheless, how Myd88 facilitates the translocation of IRF1 needs to be further elucidated. The molecular mechanisms of IRF1 nuclear translocation in foam cells challenged with TLR agonists were depicted in Figure 6K.

### IRF1 contributes to the LPS-induced aggravation of atherosclerosis

To explore the contribution of low-grade inflammation and IRF1 activation in the pathogenesis of atherosclerosis *in vivo*, ApoE^-/-^ and ApoE^-/-^ IRF1^-/-^ mice were treated with western diet for 8 weeks and intraperitoneally injected with either PBS or LPS. As determined by Oil Red O staining of the thoracoabdominal aorta and aortic sinus (Supplemental Figure 7), LPS challenge significantly increased the lipid deposition within the atherosclerotic lesions in ApoE^-/-^ mice. However, IRF1 deficiency attenuated the effects of LPS on the aggravation of atherosclerosis. Collectively, these data suggest that LPS exacerbates the development and progression of atherosclerosis in ApoE-/- mice, and these effects are dependent, at least partly, on IRF1 activation.

## Discussion

Previous studies have revealed that IRF1 plays a broad function in a variety of biology process. IRF1 expression has been documented in atherosclerotic lesions 12; however the precise effects of IRF1 in specific cell types related to atherogenesis have not been elucidated. In this study, we generated ApoE/ IRF1 double knockout mice and discovered that IRF1 deficiency not only decreased lesion area, but also induced a more stable plaque phenotype.

Foam cell formation is a hallmark of atherosclerosis development and progression, which exacerbates the disease and induces a highly characteristic architecture of vulnerable plaques 1, 3. Our data indicate an unexpected inhibitory effect of IRF1 deficiency on foam cell formation due to impaired uptake of cholesterol and improved efflux. Macrophage SRs, including SR-AI and CD36, play critical roles in foam cell formation by recognition and internalization of modified LDL 28, 29. Genetic deletion of SR-AI in atherosclerosis-prone apoE^-/-^ or LDLR^-/-^ mice significantly alleviated disease progression. Our present study clearly demonstrates that SR-AI, which we indentified as a new target gene of IRF1, might be the primary receptor responsible for IRF1-induced cholesterol uptake. For cholesterol transporters, although the levels of SR-BI and ABCA1 were both elevated when IRF1 was knocked down, the role of ABCA1 could predominate since only ABCA1 silencing abrogated the increased cholesterol efflux. However, the luciferase activities of SR-BI, ABCA1 and LOX-1 promoters were not affected by IRF1. This is probably because the significant binding sites of IRF1 have not been included in the promoter constructs, or other unknown factors could be involved in the regulation of gene expression initiated by IRF1.

It is well known that SMCs also contribute to the aggregation of foam cells 30. Surprisingly, lipid accumulation was also reduced in IRF1-silenced VSMCs, and the expression changes of genes related to cholesterol metabolism were generally consistent with changes observed in macrophages. Of note, the expression of another scavenger receptor, SCARF1, was also inhibited by IRF1 knockdown, while expression of LDLR and VLDLR were increased. More research is needed to elaborate the meaning of these changes in gene expression of various cell types in foam cell formation.

Metabolic diseases, such as atherosclerosis, nonalcoholic fatty liver (NAFL) and type 2 diabetes, were more likely to be complicated with subclinical endotoxemia 31, maybe due to increased transport of endotoxin derived from disturbed intestinal flora 32. Macrophages activated by modified lipoproteins and threshold level of endotoxin are particularly relevant to atherosclerosis development. Our current data demonstrate that LPS, which cooperates with ox-LDL and induces IRF1 activation, exacerbates the pathogenesis of atherosclerosis in ApoE^-/-^ mice. Specifically, the levels of IRF1, in both the cytoplasm and the nucleus, were elevated upon ox-LDL stimulation, and threshold levels of LPS were shown to induce translocation of IRF1 to the nucleus, followed by activation of target genes involved in cholesterol metabolism. Mechanistically, LPS stimulation led to a dramatic shift of the IRF1-Ubc9 complex to IRF1-Myd88 complex. Ubc9 is a unique SUMO E2-conjugating enzyme responsible for substrate recognition 27. It is well understood that SUMO conjugates with its substrates through activation of the (E1) enzyme [SUMO activating enzyme subunit (SAE)1/SAE2] and a conjugating (E2) enzyme (Ubc9). SUMOylation has great significance in the regulation of various cellular processes, such as protein localization, gene transcription, chromosome segregation, and DNA repair 33. It has been reported that Ubc9 is essential for IRF1 SUMOylation 34. Although the level of IRF1 was elevated upon ox-LDL stimulation, it was SUMOylated via the association with Ubc9, and trapped in the cytoplasm. LPS challenge led to the formation of a distinct protein complex containing Myd88. Myd88 is the major TLR4 intracellular adaptor molecule required for signal transduction following LPS challenge. Activated Myd88 binds to IRF1 and disrupts its interaction with Ubc9, thereby restraining IRF1 SUMOylation and promoting its nuclear translocation. It is interesting to note that other TLR agonists, such as Pam_3_CSK_4_ (TLR2 agonist), also led to IRF1 translocation and lipid accumulation in macrophages incubated with ox-LDL. This is consistent with the fact that all TLRs, except for TLR3, require MyD88 for their downstream signaling 35.

Vulnerable plaques contain fewer SMCs and less collagen in fibrous caps than stable plaques, and increased apoptotic SMCs are viewed as a hallmark of plaque instability 21. Here, we also observed a significant increase in fibrous cap thickness and collagen content in ApoE^-/-^IRF1^-/-^ mice, which correlated with increased growth and inhibited apoptosis of SMCs. Studies in primary VSMCs *in vitro* confirmed these results and indicated that IRF1 is a negative regulator of VSMC proliferation and migration, and increases its susceptibility to apoptosis. The anti-proliferative and pro-apoptotic mechanisms initiated by IRF1 have been partially characterized previously in different cell systems. Consistent with our current study, Wessely *et al.* identified IRF1 as a potential inhibitor of the proliferation and migration of the coronary artery smooth muscle cells (CASMC), as well as in neointima formation *in vivo* in a murine model of neointimal hyperplasia 36. They also explained a mechanism that IRF1 led to G1 cell cycle arrest and induced the CDK inhibitor p21. On the other hand, Zhang* et al.* proved that IRF1 has bi-directional effects on high glucose-induced proliferation of VSMCs through different regulation of cell cycle related proteins 37. Whether our results share common mechanisms with previous studies or involve some novel factors remains elusive, and requires our further study.

Compromised endothelial function is a hallmark of early atherosclerosis 38. Notably, IRF-1 has been documented to play an essential role in regulating vascular cell adhesion molecule 1 (VCAM-1) expression by arterial endothelium 39. VCAM-1 is found associated with a variety of inflammatory processes and involved in the endothelial-dependent mechanisms of mononuclear cell influx 40, and these findings indirectly suggest a possible role of IRF1 in atherogenesis. However, little-to-no IRF1 immunoreactivity was detected in endothelial cells lining the vessel lumen in our study. It could be due to insufficient accuracy of the detection technique or non-uniform distribution of IRF1 gene expression. It provides an impetus for further exploration of the role of endothelial IRF1 in atherosclerosis.

It is noteworthy that the protective role of IRF1 deficiency in atherosclerosis is multifactorial, and cell type-selective genetic manipulation should be taken into account in our future studies to elaborate on cell specific effects of IRF1 in pro-atherosclerotic mice. Besides, only male mice were used in the present study because of its high success rate and good repeatability of modeling. However, the cardiovascular characteristics, such as blood pressure, serum lipid profile, endothelial function, and abundance of thrombotic plaques of female sex are different from male sex 41, and many, but not all, of these phenotypes have been linked to the antiatherogenic effects of estrogen 42. Whether the results are consistent between different genders remains unknown and needs more study.

In summary, our study identifies a prominent role of IRF1 in the development, progression and instability of atherosclerotic lesions, and provides a mechanism explaining the link between subclinical endotoxemia and atherosclerosis, which is partly attributable to the acceleration of foam cell formation induced by nuclear translocation of IRF1 and activation of genes implicated in cholesterol metabolism. These findings provide an impetus for further exploration of the roles of IRF1 in other metabolic or inflammatory diseases, and to consider IRF1 as a target for therapeutic intervention in cardiovascular disease.

## Supplementary Material

Supplementary methods and figures.
