# Supplementary material for: Absence of Interferon Regulatory Factor 1 Protects Against Atherosclerosis in Apolipoprotein E-Deficient Mice
Source: Theranostics. 2019 Jul 9;9(16):4688–703. doi: 10.7150/thno.36862 (PMC6643443; doi:10.7150/thno.36862)
Supplement: Supplementary file 1 — Supplementary methods and figures. [file thnov09p4688s1.pdf]

SUPPLEMENTAL MATERIAL

Absence of Interferon Regulatory Factor 1 Protects against Atherosclerosis in Apolipoprotein E–Deficient Mice

Meng Du, Xiaojing Wang, Xiaoxiang Mao, Liu Yang, Kun Huang, Fengxiao Zhang, Yan Wang, Xi Luo, Cheng Wang, Jiangtong Peng, Minglu Liang, Dan Huang, Kai Huang

Supplemental Methods

Human atherosclerotic tissues

12 human atherosclerotic lesions were collected from patients undergoing carotid endarterectomy at Wuhan Union Hospital. 10 internal mammary arteries obtained from patients undergoing coronary artery bypass surgery were used as non-atherosclerotic control arteries. Written informed consent was obtained from all participants according to the declaration of Helsinki. The investigations were approved by the Ethical Committee of Huazhong University of Science and Technology.

Animals

IRF1 global knockout mice (IRF1$^{-/-}$ on a C57BL/6 background) were kindly provided by Dr Hongliang Li (Wuhan University, Wuhan, China) [1]. To obtain the ApoE$^{-/-}$IRF1$^{-/-}$ mice, we generally crossbred the IRF1$^{-/-}$ with ApoE$^{-/-}$ to get ApoE$^{+/-}$IRF1$^{+/-}$ heterozygous mice. ApoE$^{-/-}$IRF1$^{-/-}$ mice and the control ApoE$^{-/-}$ littermates were obtained by inbreeding between ApoE$^{+/-}$IRF1$^{+/-}$ heterozygous mice. The 6-week-old ApoE$^{-/-}$ and ApoE$^{-/-}$IRF1$^{-/-}$ male mice were treated with western diet (WD) for 16 weeks. For LPS-induced endotoxemia model, the 6-week-old ApoE$^{-/-}$ and ApoE$^{-/-}$IRF1$^{-/-}$ male mice were treated with western diet for 8 weeks. During the feeding period, mice were intraperitoneally injected with either PBS or LPS (5 ng per kg body weight) every 3 days (n=8 for each group) [2]. At the end of the study, Mice were euthanized via intraperitoneal injection of pentobarbital (150 mg/kg). Body weight and serum data were collected at the initiation of the study and at the time of euthanization. All the procedures involving mouse experiments were approved by the Ethics Committee of Union Hospital, Huazhong University of Science and

Technology, China, and were conducted in accordance with the National Institutes of Health (NIH) Guide for the Care and Use of Laboratory Animals.

Immunofluorescence

Immunofluorescence on human carotid artery sections and mice aortic sinus sections was performed. Briefly, for double-staining, sections were incubated with anti-IRF1 antibody (ab186384, Abcam), together with anti-CD68 antibody (ab31630, Abcam), anti-F4/80 antibody (ab6640, Abcam), anti-α-SMA antibody (ab7817, Abcam) or anti-von Willebrand Factor antibody (ab11713, Abcam) at 4℃ overnight, followed by a 30-minute incubation with secondary antibody conjugated to AlexaFluor 568 (red) and AlexaFluor 488 (green) (Molecular probes, Inc., Eugene, OR). The signals of individual and merged images for antigen detection were performed using a fluorescence microscope (Olympus, Japan) and Axiovision 4.8 software**.**

Histological analysis and quantification of atherosclerotic lesions

Mice were fasted for 4 hours and then anesthetized. Lipid accumulation of thoracoabdominal aorta was determined by *en face* Oil Red O staining. Briefly, the aortas were dissected longitudinally with an extremely fine Vanna microscissor and pinned flat on a black wax surface with 0.2-mm-diameter stainless steel pins. The pinned aortas were stained with Oil Red O and images were captured with a standard digital camera. For the microscopic evaluation of the aortic sinus lesions, hearts were fixed in 4% paraformaldehyde and cryopreserved in 15% sucrose, and then 30% sucrose. After embedded in OCT compound (Sakura Finetek, Torrance, CA), hearts were cryosectioned and 6-μm sections were collected at 80-μm intervals, starting at a 100-μm distance from the appearance of the aortic valves. Aortic sinus sections were stained for lipid accumulation with Oil red O, for morphology with hematoxylin and eosin (H&E), for collagen content with Masson's trichrome staining (Masson), for elasticfibers with elastica van Gieson staining (EVG). The relative content of macrophages and smooth muscle cells were detected by immunohistochemistry. Frozen serial sections were treated with 0.3% H_2O_2 in PBS to block endogenous peroxidase activity, followed by blocking in 4% BSA (Sigma). Primary antibodies were specific for the followings: F4/80 (ab100790, Abcam), α-SMA (ab7817, Abcam), SR-AI (ab217843, Abcam) and ABCA1 (ab66217, Abcam). All sections were stained with biotinylated secondary antibodies and detected using ABC reagents (Vector

Laboratories). Collected images were quantitated by quantitative morphometry using the Image Pro Plus program. The lesion areas were determined by calculating the mean lesion area of the four sections at 80-μm intervals. For evaluation of the relative content of the stained constituents, we determined the percentage of blue (collagen on Masson's trichrome staining) and DAB-positive (immunohistochemistry) areas to the total plaque areas. Fibrous cap area was quantified as a percent of total plaque area. We defined fibrous caps as the VSMC and proteoglycan-rich area overlying the cholesterol-rich, matrix-poor, acellular regions of the necrotic cores [3]. Elastic lamina destruction of the tunica media was evaluated by quantifying the ruptures (i.e. discontinuities or fractures) of elastic fibers [4].

Generation of recombinant adenovirus

Replication-defective recombinant adenovirus carrying the entire coding sequence of IRF1 (AdIRF1) was constructed with Adenovirus Expression Vector Kit (Takara Bio Inc., Kusatsu, Japan). An adenovirus-only-containing green fluorescence protein (GFP) was used as a negative control (AdGFP). To generate adenovirus expressing shRNA against IRF1 (AdshIRF1), three siRNAs were designed and the one with the optimal knockdown efficiency was chosen to create shRNA and then recombined into adenoviral vectors. The negative control adenovirus was designed to express non-targeting "Universal Control" shRNA (AdshRNA). Amplification and purification of recombinant adenovirus was performed according to the manufacturer's instructions (Takara Bio).

RNA extraction and qRT-PCR

Total RNA was extracted from cells with the use of TRIzol reagent (D9108A, Takara Bio). RNA was reverse-transcribed using the RNA PCR Kit (RR036A, Takara Bio). Quantitative polymerase chain reaction (PCR) amplification was performed with an ABI PRISM 7900 Sequence Detector system (Applied Biosystem, Foster City, CA), according to the manufacturer's instructions. Relative gene expression (normalized to endogenous control gene β-actin) was calculated using the comparative Ct method formula $2^{-\Delta\Delta Ct}$. The real-time PCR primer sequences are shown in Supplementary Table 1.

Western blot

Cells or tissues were harvested at indicated times and homogenized in ice-cold suspension buffer

supplemented with a proteinase inhibitor cocktail (Sigma-Aldrich). Protein concentrations were determined using the BCA Protein assay kit (Thermo Scientific, Waltham, MA). Equal amounts of protein were fractionated by SDS polyacrylamide gels, followed by immunoblotting with the following primary antibodies: anti-SR-AI antibody (ab217843, Abcam), anti-IRF1 antibody (ab186384, Abcam), anti-Ubc9 antibody (ab21193), anti-MyD88 antibody (ab28763), and anti-Sumo1 antibody (sc-130275, Santa Cruz). Membranes were then incubated with peroxidase-conjugated secondary antibody, and specific bands were detected with a Bio-Rad (Hercules, CA) imaging system.

Immunoprecipitations and Mass Spectrometry

Immunoprecipitation was performed to determine protein complex formation. Cells were washed with cold PBS and lysed with lysis buffer (20mM Tris–HCl, pH 7.4, 150mM NaCl, 1mM EDTA, 1% Triton X-100) containing Protease Inhibitor Cocktail Tablets (04693132001, Roche). After being pre-cleared with normal immunoglobulin G and protein A/G-agarose beads (11719394001 and 11719386001, Roche), lysates were subjected to immunoprecipitation with specific primary antibody overnight, and protein A/G–agarose beads were added before samples were rotated at 4°C for a further 2 hour. Coimmunoprecipitated proteins were washed five times with lysis buffer and separated with SDS–PAGE, followed by mass spectrometry or Western blot.

GST pulldown assays

Purified GST or GST-tagged IRF1 proteins were individually mixed with glutathione agarose beads at room temperature for 30 min. The beads were pelleted by centrifugation and washed with binding buffer containing 20 mM HEPES (pH 7.5), 100 mM NaCl, 5 mM $MgCl_2$, 1 mM DTT, and 0.05% Tween 20. The resulting immobilized proteins were then incubated with His-tagged Ubc9 or Myd88 at room temperature for 30 min. Beads were pelleted and washed 5 times with wash buffer containing 50 mM HEPES (pH 7.5), 100 mM NaCl, 5 mM $MgCl_2$, 1 mm DTT, and 0.05% Tween 20. The bound proteins were eluted from the beads and analyzed by western blotting.

Luciferase assays

Promoters of SR-AI, CD36, LOX-1, SR-BI, ABCA1 and ABCG1 were PCR amplified and subcloned into the pGL3-Basic vector (Promega, Madison, WI) using the One Step Cloning Kit (C112-02,

Vazyme Biotech Ltd., Nanjing, China). Luciferase reporter constructs were co-transfected with an internal control plasmid pRL-TK (Renilla luciferase reporter plasmid, Promega) into HEK293T cells, followed by infection with AdGFP or AdIRF1. Then cells were harvested, lysed, and the luciferase activity was determined with Dual Luciferase Reporter Assay Kit (Promega) according to the manufacturer's instruction.

Chromatin immunoprecipitation (ChIP) assay

ChIP assay was performed according to the instructions (CHIP assay kit, Millipore) using monoclonal antibodies against IRF1. DNA samples recovered after immunoprecipitation were subjected to PCR. As negative controls, ChIP was performed in the presence of IgG.

Foam cell formation assay and quantification of cholesterol content

Macrophages were incubated with 50 μg/ml ox-LDL for different times, fixed with ethanol, and stained with Oil Red O as described [5]. Intracellular cholesterol content was measured as previously described [6]. Briefly, total lipids were extracted from cells by adding hexane: isopropanol at a 3:2 ratio. The solvent was collected to measure total cholesterol using the Cholesterol/Cholesteryl Ester Quantification Kit II (Biovision, Milpitas, CA). Values of total cholesterol were normalized to the total protein content of extracts.

Ox-LDL uptake assay

The internalization of ox-LDL by macrophages or VSMCs was performed using ox-LDL labeled with fluorescent probe, 1,1'-dioctadecyl-1 to 3,3,3',3'- tetramethylindocarbocyanine perchlorate (Dil, Biomedical Technologies, inc) as described previously [7]. After specified culture conditions, cells were extensively washed and incubated with 10 μg/ml Dil-ox-LDL for 4 hours at 37 °C. The uptake of Dil-ox-LDL was determined by flow cytometry.

Cholesterol efflux assay

To assess NBD-cholesterol efflux, macrophages or VSMCs were incubated in phenol red-free RPMI 1640 medium containing 5 μmol/l NBD-cholesterol (Cayman Chemical, Ann Arbor, MI) for 6 h at 37˚C. Following incubation, cells were washed with PBS three times and were then incubated with

HDL or apoAI, as lipid acceptors. Subsequently, the cells were harvested after 4 h, and the medium and cell lysate were collected for the detection of fluorescence. Cholesterol efflux was expressed as percent fluorescence in medium relative to total fluorescence.

VSMC migration assay

With the modified Boyden chamber method, SMC migration is determined in Transwell cellculture chambers with 8 mm pores. VSMCs (5×10^4 cells per well) suspended in 100 μl of DMEM was added to the upper chamber. Migration was induced by the addition of 10% FBS in DMEM to the lower compartment. After 5 hours of incubation, cells on both side of the membrane were fixed and stained with 1% toluidine blue (Sigma-Aldrich). Cells on the upper side of the membrane were removed with a cotton swab. The average number of cells from five randomly chosen high power (200X) fields on the lower side of the membrane was counted.

VSMC Tunel assay

The Tunel assay was performed with a Promega (Madison, WI) kit. VSMCs were cultured on glass coverslips in a 12-well plate at a density of 2×10^5 cells/well. After ox-LDL treatment, VSMCs were washed with PBS and fixed in 4% paraformaldehyde for 10 min at room temperature. The fixed cells were then incubated for 2 min on ice in a permeabilization solution (0.1% Triton X-100 in 0.1% sodium citrate). The slides were rinsed with PBS before the rTdT incubation mixture was added. After incubation for 60 min at 37 °C, the slides were rinsed three times with 2× SSC, and the nuclei were counterstained with 4′,6′-diamino-2-phenylindole. The numbers of Tunel-positive cells were counted in five randomly chosen fields (magnification, ×200), and the mean and S.E. were calculated.

Cell culture

Peritoneal macrophages were isolated from C57BL/6 mice [8]. Briefly, the peritoneal cavity was first lavaged with sterile NaCl (0.9%), and then the lavage fluids were collected, pooled and centrifuged. Cell pellets were suspended in RPMI1640 medium (Gibco) supplemented with 10% FBS (Gibco). Macrophages were allowed to adhere in culture plates for 2 h. Non-adherent cells were removed by washing and the adherent cells were maintained for 24 h in 10% serum-containing medium for further study. Primary SMCs were obtained from 6 to 8-week-old C57 male mouse aortas using collagenase-

elastase digestion as follows [9]: aortas were excised, washed in phosphatebuffered saline, and incubated in DMEM containing 1 mg/mL of Collagenase type II (Worthington Biochemical Corp) for 10 to 15 min. Then, under microscopic guidance, the vessels were stripped of adventitia, minced with scissors and digested with Collagenase 0.5 mg/ml (Sigma type I, C-0130) and Elastase 0.125 mg/ml (Sigma type III, E-0127) in serum-free DMEM with antibiotics at 37℃ until most cells were in suspension. The cell suspension was recovered and centrifuged at 400g for 5 min, then resuspended in DMEM (Invitrogen) with 20% fetal bovine serum (FBS, HyClone), 2% penicillin–streptomycin, and cultured in plates. The cells used in the experiments were from passages 3 to 5. HEK293T cells (CRL-11268), THP-1 cells (TIB-202) and T/G HA-VSMC (CRL-1999) were obtained from ATCC and cultured according to the manufacturer's instructions. All of the cell lines were free of mycoplasma contamination (tested by the vendors using the MycoAlert kit from Lonza). No cell lines used in this study are found in the database of commonly misidentified cell lines (ICLAC and NCBI Biosample) based on short tandem repeats (STR) profiling performed by vendors.

Statistical analysis

GraphPad Prism software (GraphPad Software Inc., La Jolla, CA) was used for statistical analyses. Band intensity in western blot images was quantified with Image J Software. Values are expressed as means ± SEM of at least three independent experiments. Student's t test was used to assess the statistical significance of the differences between two groups. Benjamini–Hochberg corrections for multiple variables were used in transcriptional studies. For multiple groups, significance was evaluated by one-way ANOVA with Bonferroni test (homogeneity of variance) or Tamhanes's T2 test (heterogeneity of variance). $P < 0.05$ was considered statistically significant. Randomization and blinding strategy was used whenever possible. Animal cohort sizes were determined on the basis of similar previous studies.

References

1. Jiang DS, Li L, Huang L, Gong J, Xia H, Liu X, et al. Interferon regulatory factor 1 is required for cardiac remodeling in response to pressure overload. Hypertension 2014; 64: 77-86.

2. Geng S, Chen K, Yuan R, Peng L, Maitra U, Diao N, et al. The persistence of low-grade inflammatory monocytes contributes to aggravated atherosclerosis. Nat Commun 2016; 7: 13436.

3. Clarke MC, Figg N, Maguire JJ, Davenport AP, Goddard M, Littlewood TD, et al. Apoptosis of vascular

smooth muscle cells induces features of plaque vulnerability in atherosclerosis. Nat Med 2006; 12: 1075-80.
4. de Nooijer R, Bot I, von der Thusen JH, Leeuwenburgh MA, Overkleeft HS, Kraaijeveld AO, et al. Leukocyte cathepsin S is a potent regulator of both cell and matrix turnover in advanced atherosclerosis. Arterioscler Thromb Vasc Biol 2009; 29: 188-94.
5. Furuhashi M, Tuncman G, Gorgun CZ, Makowski L, Atsumi G, Vaillancourt E, et al. Treatment of diabetes and atherosclerosis by inhibiting fatty-acid-binding protein aP2. Nature 2007; 447: 959-65.
6. Robinet P, Wang Z, Hazen SL, Smith JD. A simple and sensitive enzymatic method for cholesterol quantification in macrophages and foam cells. J Lipid Res 2010; 51: 3364-9.
7. Ouchi N, Kihara S, Arita Y, Nishida M, Matsuyama A, Okamoto Y, et al. Adipocyte-derived plasma protein, adiponectin, suppresses lipid accumulation and class A scavenger receptor expression in human monocyte-derived macrophages. Circulation 2001; 103: 1057-63.
8. Ray A, Dittel BN. Isolation of mouse peritoneal cavity cells. J Vis Exp 2010.
9. Golovina VA, Blaustein MP. Preparation of primary cultured mesenteric artery smooth muscle cells for fluorescent imaging and physiological studies. Nat Protoc 2006; 1: 2681-7.

Supplemental Table 1. qRT-PCR primers used

Gene	Forward primer	Reverse primer
SR-A Ⅰ	TGGAGGAGAGAATCGAAAGCA	CTGGACTGACGAAATCAAGGAA
SR-A Ⅱ	CCTCCAGGGACTTACGGGT	CCAGTGAGACCTATGTCACCT
SR-B Ⅰ	TTTGGAGTGGTAGTAAAAAGGGC	TGACATCAGGGACTCAGAGTAG
SR-B Ⅱ	AGAAGGCGGTAGACCAGAC	CTGTAGGTGTATGGCCCCAC
LOX-1	CAAGATGAAGCCTGCGAATGA	ACCTGGCGTAATTGTGTCCAC
SCARF1	TGGGACTAGAGCTGGTGTTCT	CAGATGGGGATGGTGCATTCT
VLDLR	GAGTCTGACTTCGTGTGCAAA	GAACCGTCTTCGCAATCAGGA
LDLR	TCAGACGAACAAGGCTGTCC	CCATCTAGGCAATCTCGGTCTC
ABCA1	GCTTGTTGGCCTCAGTTAAGG	GTAGCTCAGGCGTACAGAGAT
ABCG1	GTGGATGAGGTTGAGACAGACC	CCTCGGGTACAGAGTAGGAAAG
CD36	ATGGGCTGTGATCGGAACTG	TTTGCCACGTCATCTGGGTTT
PPARα	AACATCGAGTGTCGAATATGTGG	CCGAATAGTTCGCCGAAAGAA
PPARγ	GGAAGACCACTCGCATTCCTT	GTAATCAGCAACCATTGGGTCA
LXRα	CTGATTCTGCAACGGAGTTGT	GACGAAGCTCTGTCGGCTC
LXRβ	GCCTGGGAATGGTTCTCCTC	AGATGACCACGATGTAGGCAG
IRF1	GGCCGATACAAAGCAGGAGAA	GGAGTTCATGGCACAACGGA

Supplemental Table 2. Plasma lipid profiles and body weights of ApoE$^{-/-}$ and ApoE$^{-/-}$IRF1$^{-/-}$ mice fed western diet.

	**Western Diet**	
	**ApoE$^{-/-}$ (n=12)**	**ApoE$^{-/-}$IRF1$^{-/-}$ (n=12)**
Total cholesterol (mg/dl)	1001.3±173.3	931.4±143.2
Triglycerides (mg/dl)	90.3±19.6	82.8±18.4
LDL cholesterol (mg/dl)	604.5±51.2	584.4±69.6
HDL cholesterol (mg/dl)	55.5±6.9	51.3±6.1
Body weight (g)	31.3±3.6	29.9+3.1

LDL, low-density lipoprotein; HDL, high-density lipoprotein

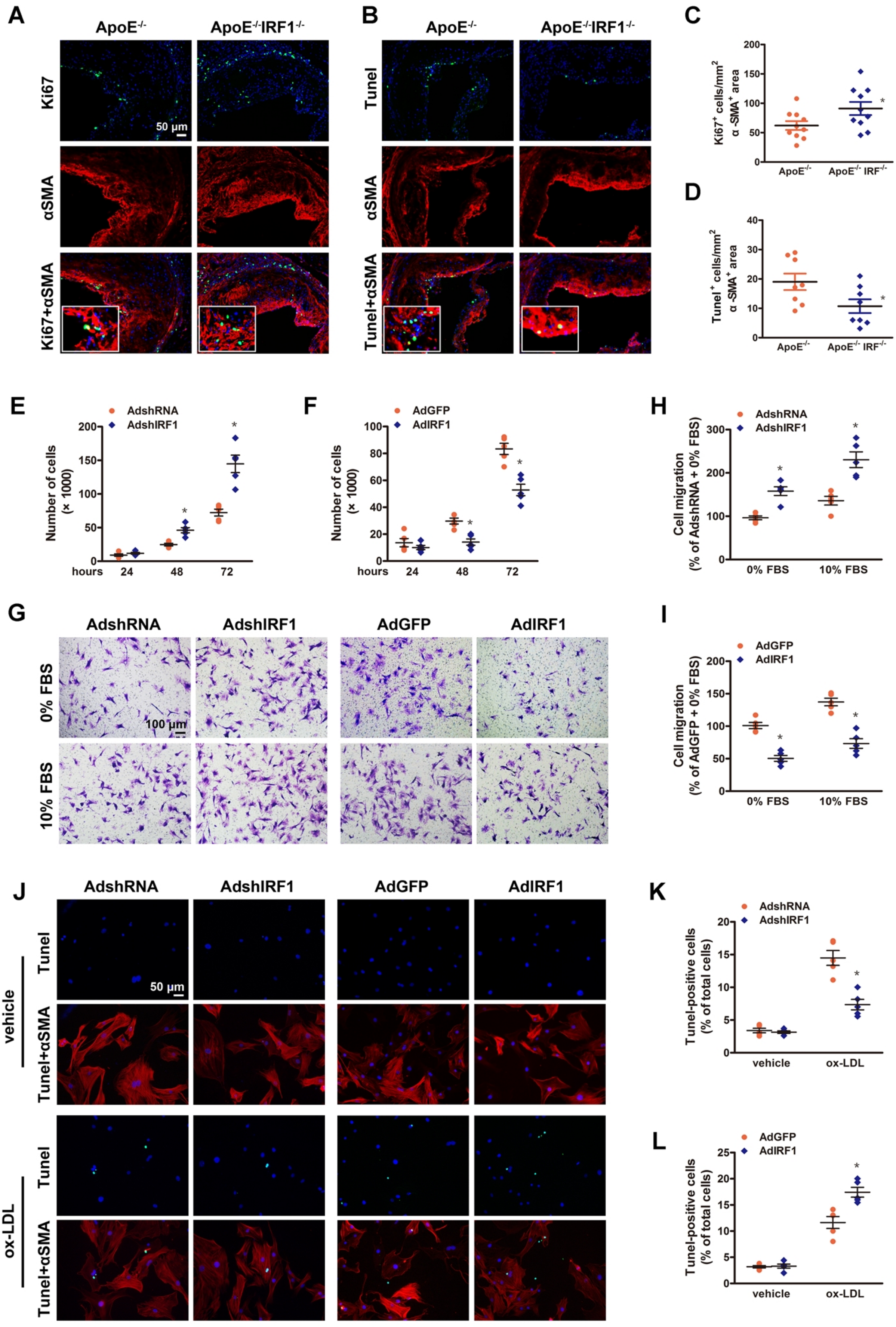

A
ApoE-/-
ApoE-/-IRF1-/-
Ki67
αSMA
Ki67+αSMA
50 μm
B
Tunel
Tunel+αSMA
C
Ki67+ cells/mm2 α -SMA+ area
ApoE-/- IRF-/-
D
Tunel+ cells/mm2 α -SMA+ area
E
AdshRNA
AdshIRF1
Number of cells (× 1000)
hours
F
AdGFP
AdIRF1
G
0% FBS
10% FBS
100 μm
H
Cell migration (% of AdshRNA + 0% FBS)
I
Cell migration (% of AdGFP + 0% FBS)
J
vehicle
ox-LDL
K
Tunel-positive cells (% of total cells)
L

Supplemental Figure 1. IRF1 deficiency promotes proliferation and inhibits apoptosis of VSMCs. A and C, Images show Ki67 staining (green) of nuclei in sections of aortic sinus from ApoE$^{-/-}$ and ApoE$^{-/-}$IRF1$^{-/-}$ mice, and the number of proliferating cells was quantified in VSMC-rich areas (α-SMA staining, red). Scale bar = 50 μm. Data are expressed as mean ± SEM (n=10 per group). * $P < 0.05$ vs. ApoE$^{-/-}$ mice. **B and D,** Images show Tunel staining (green) of nuclei in sections of aortic sinus from ApoE$^{-/-}$ and ApoE$^{-/-}$IRF1$^{-/-}$ mice, and the number of apoptotic cells was quantified in VSMC-rich areas (α-SMA staining, red). Scale bar = 50 μm. Data are expressed as mean ± SEM (n=8 per group). * $P < 0.05$ vs. ApoE$^{-/-}$ mice. **E-L,** IRF1 expression was silenced (AdshIRF1 and control AdshRNA) or enforced (AdIRF1 and control AdGFP) in mice primary smooth muscle cells cultured in vitro. VSMC proliferation **(E, F)** was measured and quantified as viable cell number using a hemocytometer at different time points. VSMC migration **(G-I)** was examined using transwells after stimulation with serum-free DMEM (0% FBS) or 10% FBS. VSMC apoptosis **(J-L)** was evaluated by Tunel staining with or without ox-LDL challenge. Scale bar = 100 μm in panel **G**. Scale bar = 50 μm in panel **J**. Data represent the mean ± SEM of five independent experiments. Student's t test was used for data analysis. * $P < 0.05$ vs. AdshRNA group or AdGFP group.

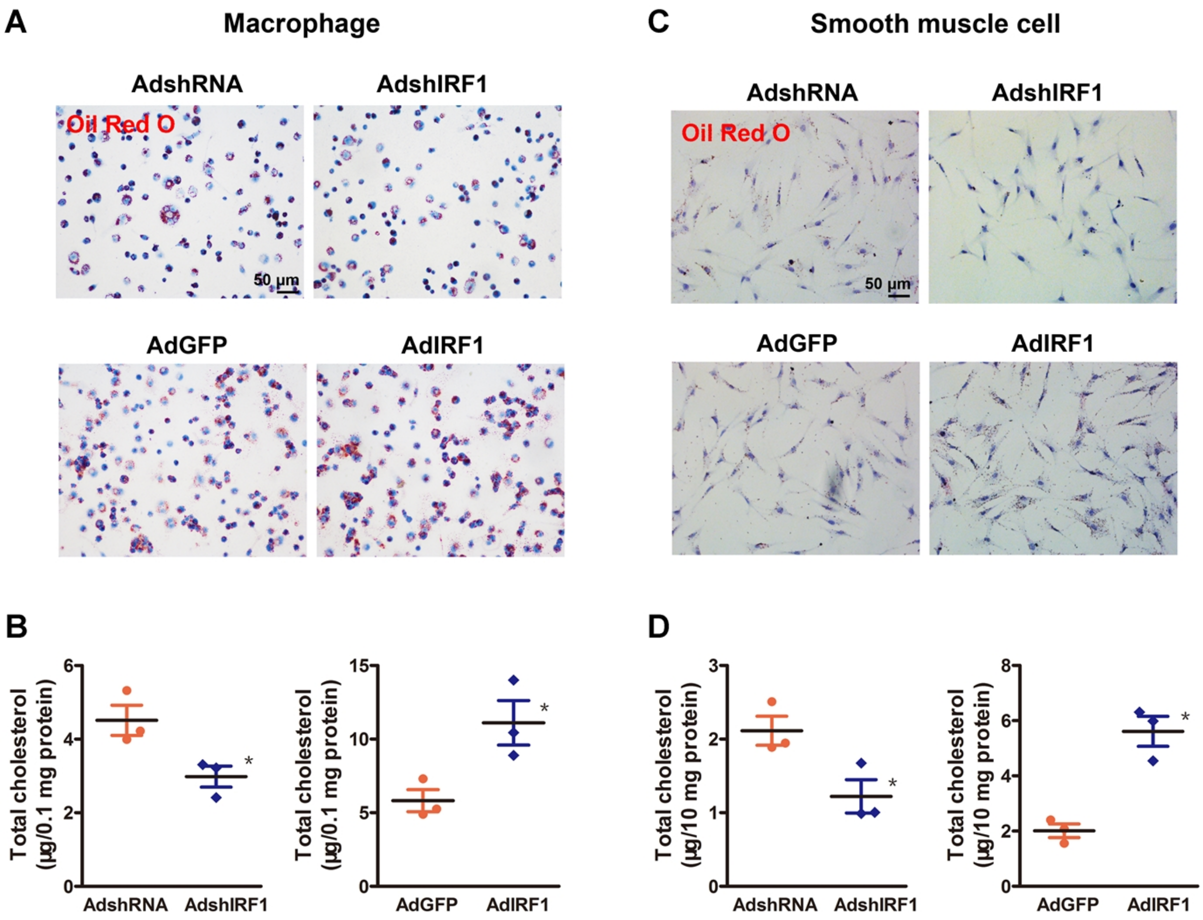

Supplemental Figure 2. IRF1 contributes to foam cell formation of human cells. IRF1 expression was silenced (AdshIRF1 and control AdshRNA) or enforced (AdIRF1 and control AdGFP) in human THP-1 macrophages and a human VSMC line (T/G HA-VSMC). **A and C,** Representative images of Oil Red O staining of macrophages **(A)** and smooth muscle cells **(C)** incubated with ox-LDL (50 μg/mL). Scale bar = 50 μm. **B and D,** Total cholesterol content was measured in foam cells derived from macrophages **(B)** and smooth muscle cells **(D)**. Data represent the mean ± SEM of three independent experiments. * $P < 0.05$ vs. AdshRNA group or AdGFP group.

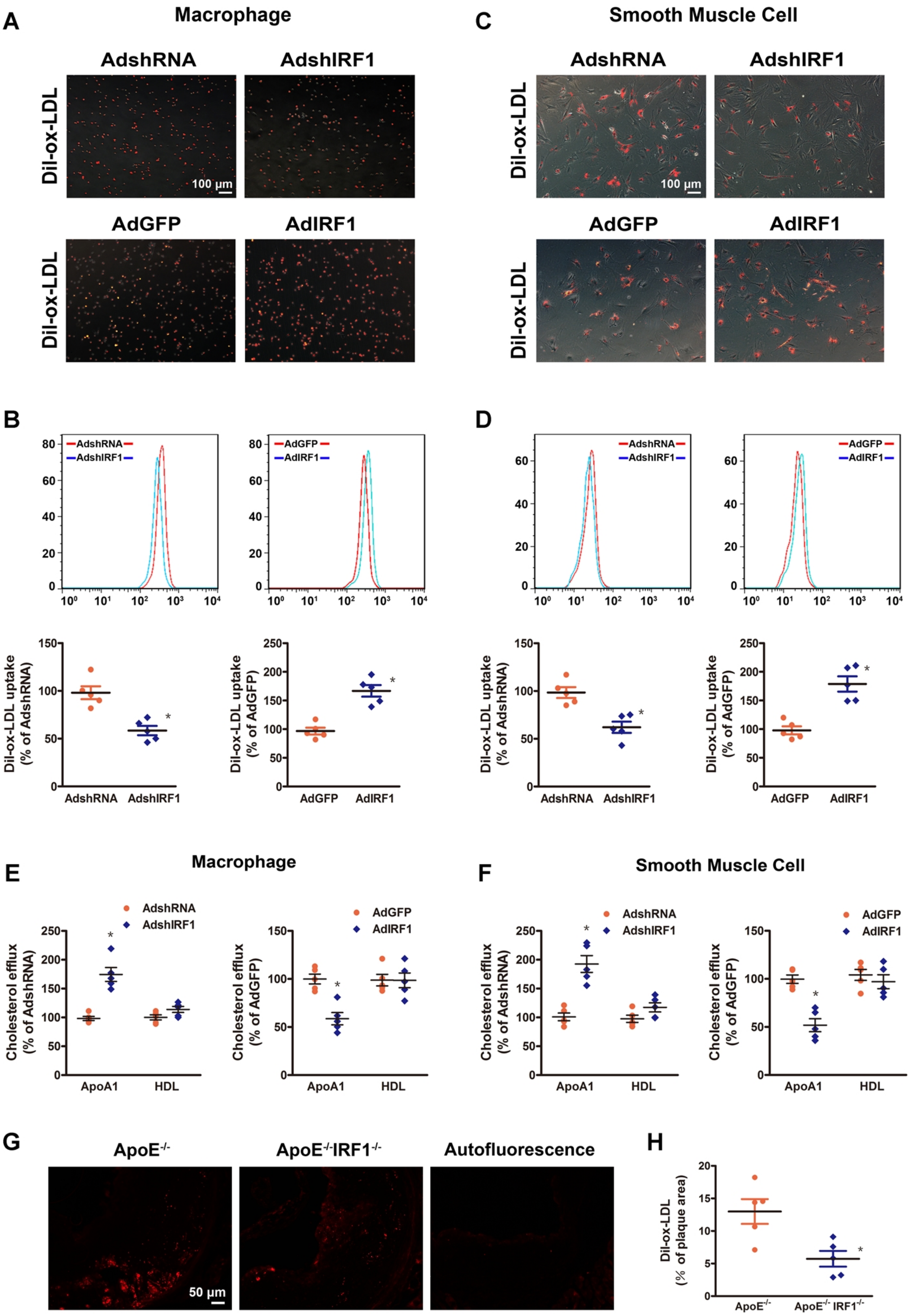

A
Macrophage
AdshRNA
AdshIRF1
Dil-ox-LDL
100 μm
AdGFP
AdIRF1
B
C
Smooth Muscle Cell
D
Dil-ox-LDL uptake (% of AdshRNA)
Dil-ox-LDL uptake (% of AdGFP)
E
F
Cholesterol efflux (% of AdshRNA)
Cholesterol efflux (% of AdGFP)
ApoA1
HDL
G
ApoE-/-
ApoE-/-IRF1-/-
Autofluorescence
50 μm
H
Dil-ox-LDL (% of plaque area)

Supplemental Figure 3. IRF1 Deficiency Inhibits the Uptake of Modified Lipoproteins and Promotes Cholesterol Efflux. IRF1 expression was silenced (AdshIRF1 and control AdshRNA) or enforced (AdIRF1 and control AdGFP) in mice peritoneal macrophages and primary smooth muscle cells cultured in vitro. **A and C,** Representative images of macrophages **(A)** and smooth muscle cells **(C)** incubated with fluorescently labeled ox-LDL (Dil-ox-LDL) for 4 h at 37°C. Scale bar = 100 μm. **B and D,** Flow cytometry analysis of Dil-ox-LDL uptake in macrophages **(B)** and smooth muscle cells **(D)**, and the results were shown as a percent of fluorescence intensity determined in AdshRNA or AdGFP group. **E and F,** Apolipoprotein A1 (ApoA1)- and high-density lipoprotein (HDL)-dependent cholesterol efflux in macrophages **(E)** and smooth muscle cells **(F)**, and the results were shown as a percent of cholesterol efflux rate determined in AdshRNA or AdGFP group. Data represent the mean ± SEM of five independent experiments. * $P < 0.05$ vs. AdshRNA group or AdGFP group. **G and H,** $ApoE^{-/-}$ and $ApoE^{-/-}IRF1^{-/-}$ mice were injected with DiI-ox-LDL. After 18 hours, sections from aortic sinus were visualized under fluorescent microscope, photographed, and quantified. Scale bar = 50 μm. Data are expressed as mean ± SEM (n=5 per group). *$P < 0.05$ vs. $ApoE^{-/-}$ mice.

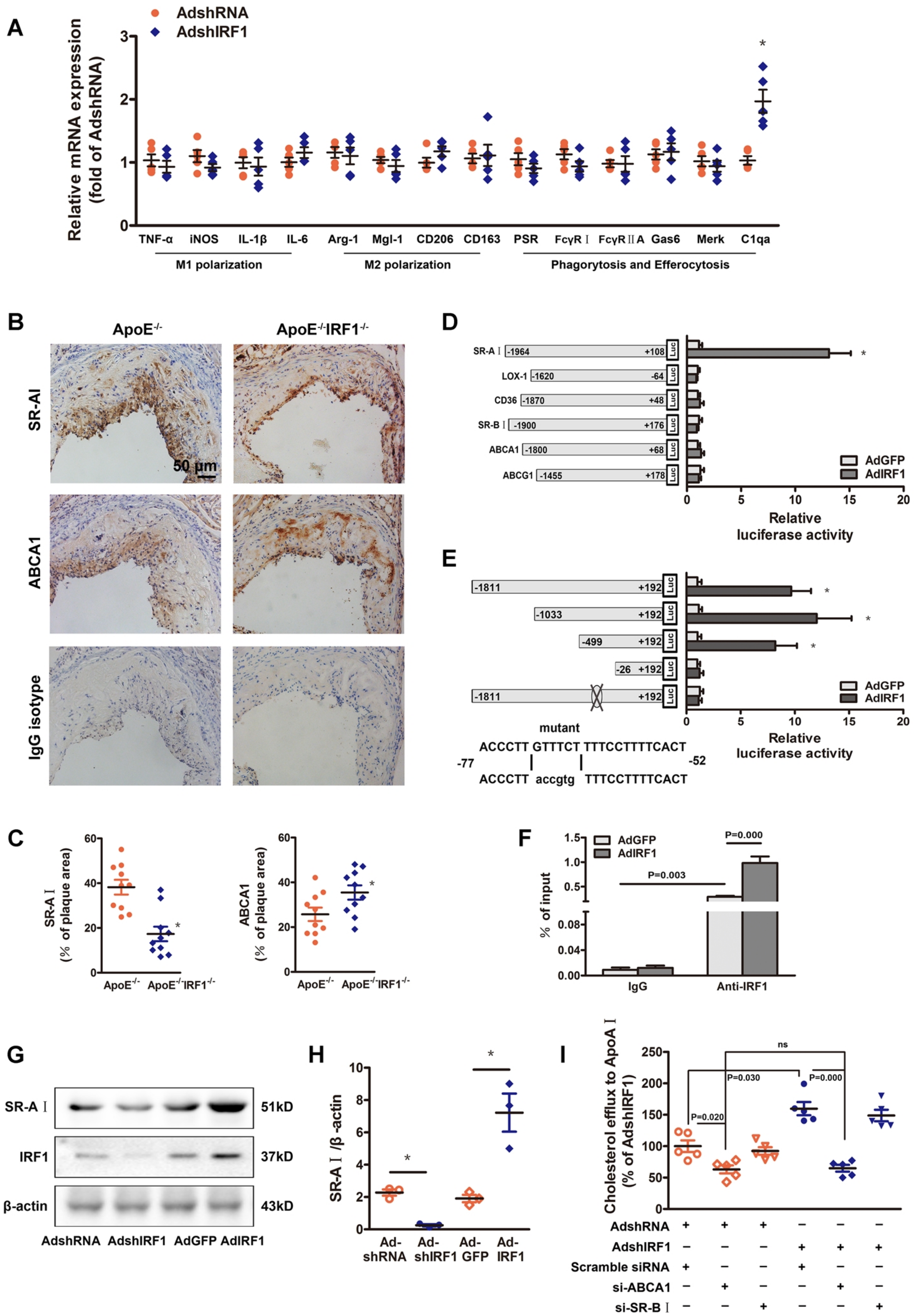
A
AdshRNA
AdshIRF1
Relative mRNA expression (fold of AdshRNA)
TNF-α iNOS IL-1β IL-6 Arg-1 Mgl-1 CD206 CD163 PSR FcγR Ⅰ FcγR Ⅱ A Gas6 Merk C1qa
M1 polarization
M2 polarization
Phagorytosis and Efferocytosis
B
ApoE-/-
ApoE-/-IRF1-/-
SR-AI
ABCA1
IgG isotype
50 μm
C
SR-A Ⅰ (% of plaque area)
ABCA1 (% of plaque area)
ApoE-/- ApoE-/-IRF1-/-
D
SR-A Ⅰ -1964 +108 Luc
LOX-1 -1620 -64 Luc
CD36 -1870 +48 Luc
SR-B Ⅰ -1900 +176 Luc
ABCA1 -1800 +68 Luc
ABCG1 -1455 +178 Luc
AdGFP
AdIRF1
Relative luciferase activity
E
-1811 +192
-1033 +192
-499 +192
-26 +192
-1811 +192
mutant
ACCCTT GTTTCT TTTCCTTTTCACT
-77 -52
ACCCTT accgtg TTTCCTTTTCACT
AdGFP
AdIRF1
Relative luciferase activity
F
AdGFP
AdIRF1
P=0.000
P=0.003
% of input
IgG
Anti-IRF1
G
SR-A Ⅰ 51kD
IRF1 37kD
β-actin 43kD
AdshRNA AdshIRF1 AdGFP AdIRF1
H
SR-A Ⅰ /β -actin
Ad-shRNA Ad-shIRF1 Ad-GFP Ad-IRF1
I
Cholesterol efflux to ApoA Ⅰ (% of AdshIRF1)
ns
P=0.030
P=0.000
P=0.020
AdshRNA + + + − − −
AdshIRF1 − − − + + +
Scramble siRNA + − − + − −
si-ABCA1 − + − − + −
si-SR-B Ⅰ − − + − − +

Supplemental Figure 4. IRF1 facilitates SR-AI expression in human cells. A, The relative mRNA levels of genes involved in polarization, phagorytosis and efferocytosis of mice peritoneal macrophages silenced with AdshIRF1 or AdshRNA. **B and C,** Cross-sections of aortic sinus from $ApoE^{-/-}$ and $ApoE^{-/-}IRF1^{-/-}$ mice were immunostained with antibodies against SR-AI and ABCA1. Staining with IgG isotype was used as the negative control. Positive stained areas were quantified as a percentage of total plaque area. Scale bar = 50 μm. Data are expressed as mean ± SEM (n=10 per group). * $P < 0.05$ vs. $ApoE^{-/-}$ mice. **D,** Luciferase reporter constructs containing murine gene promoters of SR-AI, LOX-1, CD36, SR-BI, ABCA1 and ABCG1 were co-transfected with an internal control plasmid pRL-TK into HEK293T cells, followed by infection with AdIRF1 or AdGFP. The relative luciferase activities are expressed as a percent of values determined in AdGFP group. **E,** Luciferase reporter constructs containing human SR-AI promoter truncations or its mutants were co-transfected with an internal control plasmid pRL-TK into HEK293T cells, followed by infection with AdIRF1 or AdGFP. The relative luciferase activities are expressed as a percent of values determined in AdGFP group. **F,** Chromatin immunoprecipitation (ChIP) assay revealed the affinity of IRF1 on SR-AI promoter in human THP-1 macrophages. **G,** Representative immunoblot for SR-AI and IRF1 in human THP-1 macrophages infected with AdshIRF1 or AdIRF1. **H,** Quantification of band density in panel **G**. **I,** The cholesterol efflux to ApoAI in IRF1 knocked-down macrophages silenced with si-ABCA1 or si-SR-BI. Data represent the mean ± SEM of three to five independent experiments. Benjamini–Hochberg corrections for multiple variables were used in transcriptional studies. One-way ANOVA with Bonferroni test was used to produce the *P* values given in figure. * $P < 0.05$ vs. AdshRNA group or AdGFP group. ns, no significance.

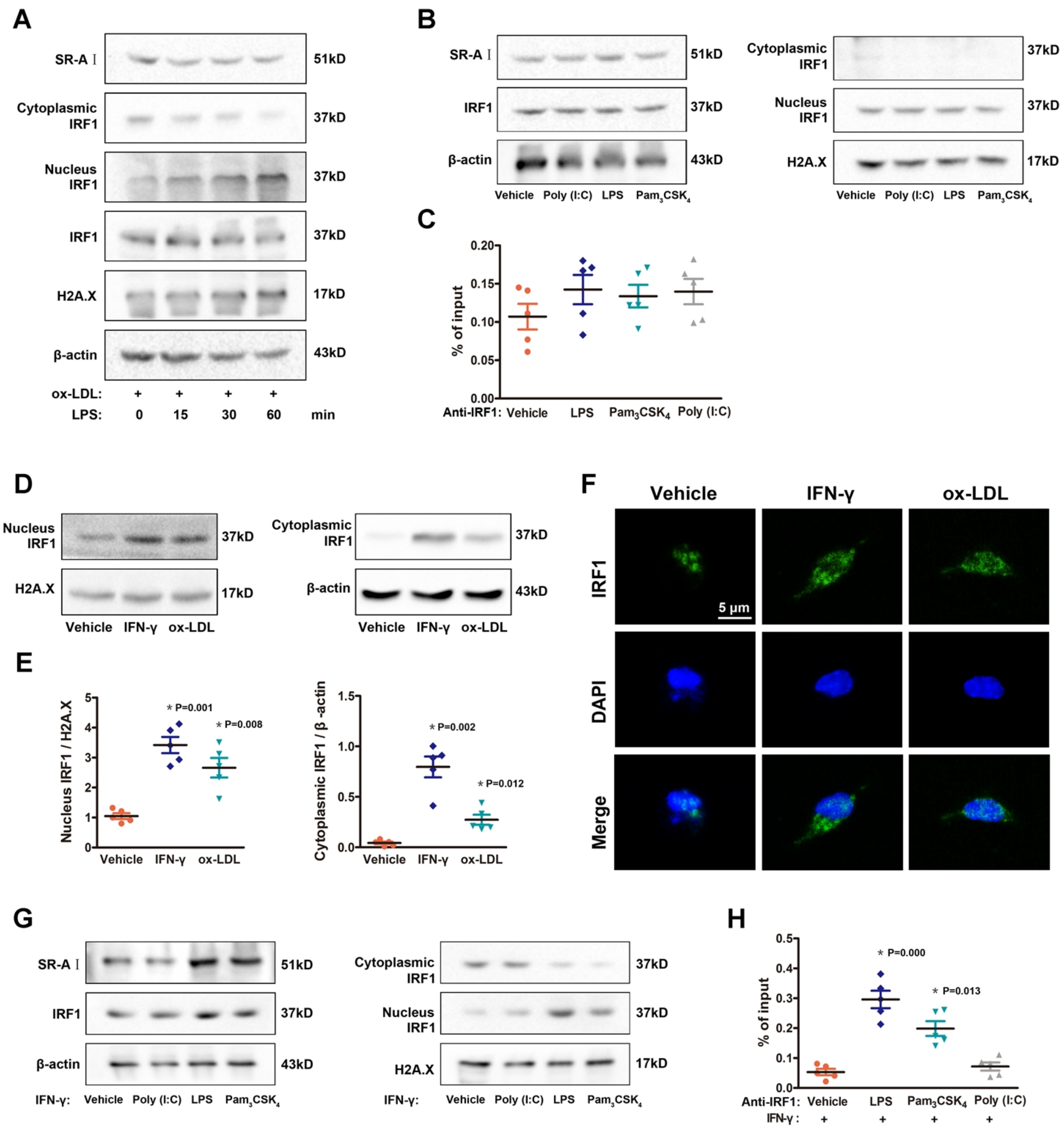

Supplemental Figure 5. LPS facilitate IRF1 nuclear translocation in macrophages incubated with ox-LDL or IFN-γ. A, Effects of LPS (50 pg/mL) on IRF1 nuclear translocation and SR-AI expression in ox-LDL incubated peritoneal macrophages at different time points, as determined by Western blot. **B,** Effects of TLR agonists on IRF1 nuclear translocation and SR-AI expression in macrophages without ox-LDL challenge, as determined by Western blot. **C,** Effects of TLR agonists on the interaction of IRF1 with SR-AI promoter in macrophages without ox-LDL challenge, as

determined by ChIP assay. **D,** Representative immunoblot for IRF1 expression in nucleus and cytoplasm of macrophages incubated with ox-LDL or IFN-γ. **E,** Quantification of band density in panel **D**. **F,** IRF1 expression in nucleus and cytoplasm of macrophages incubated with ox-LDL or IFN-γ, as determined by immunofluorescence assay. Scale bar = 5 μm. **G,** Effects of TLR agonists on IRF1 nuclear translocation and SR-AI expression in macrophages treated with IFN-γ, as determined by Western blot. **H,** Effects of TLR agonists on the interaction of IRF1 with SR-AI promoter in macrophages treated with IFN-γ, as determined by ChIP assay. Data represent the mean ± SEM of five independent experiments. One-way ANOVA with Bonferroni test was used to produce the *P* values given in figure. * $P < 0.05$ vs. vehicle group.

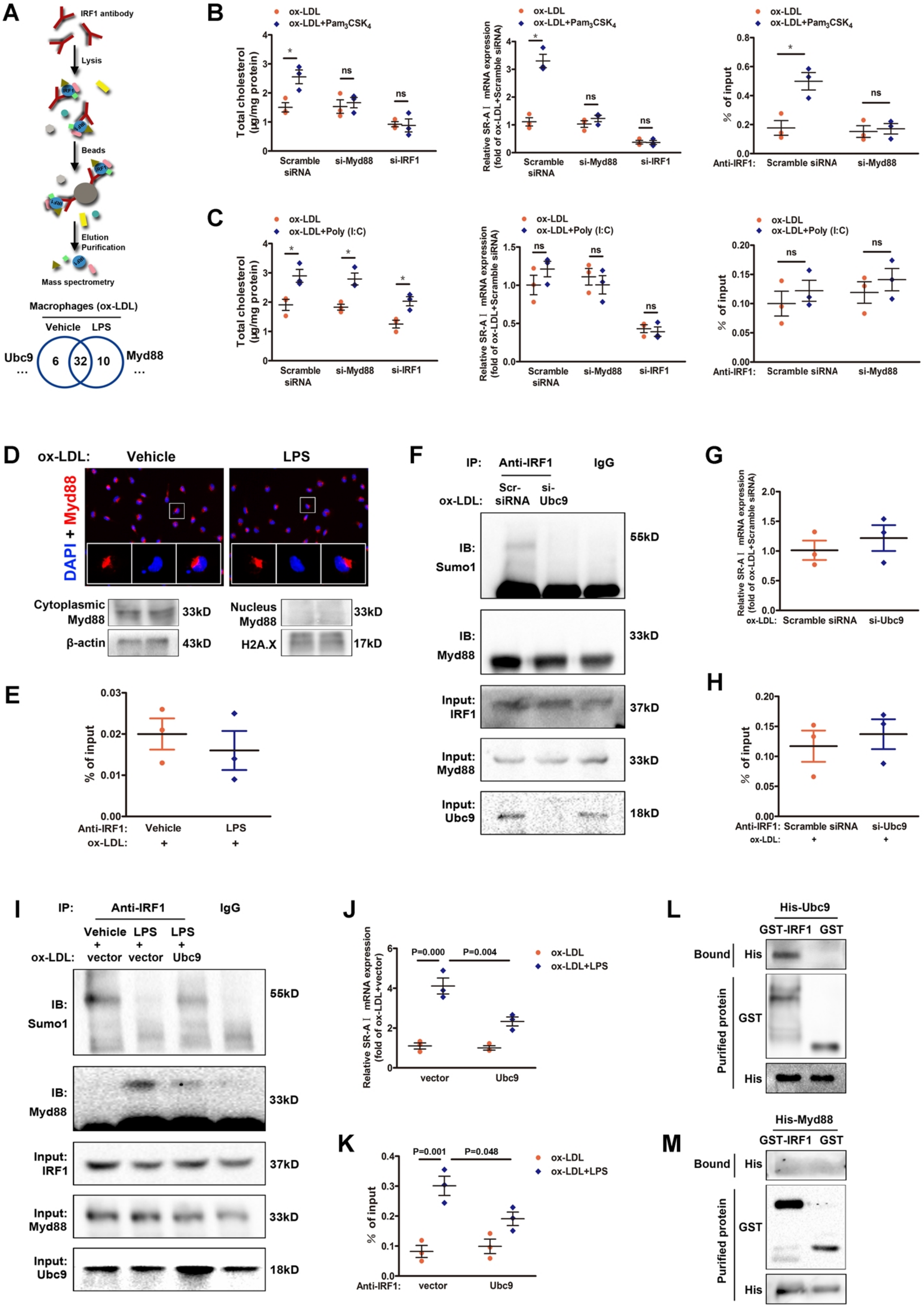
A
IRF1 antibody
Lysis
Beads
Elution
Purification
Mass spectrometry
Macrophages (ox-LDL)
Vehicle LPS
Ubc9
6 32 10
Myd88
B
ox-LDL
ox-LDL+Pam3CSK4
Total cholesterol (μg/mg protein)
Relative SR-A Ⅰ mRNA expression (fold of ox-LDL+Scramble siRNA)
% of input
Scramble siRNA
si-Myd88
si-IRF1
Anti-IRF1:
C
ox-LDL+Poly (I:C)
D
ox-LDL:
Vehicle
LPS
DAPI + Myd88
Cytoplasmic Myd88
β-actin
Nucleus Myd88
H2A.X
33kD
43kD
17kD
E
Anti-IRF1:
F
IP: Anti-IRF1 IgG
Scr-siRNA
si-Ubc9
IB: Sumo1
IB: Myd88
Input: IRF1
Input: Myd88
Input: Ubc9
55kD
37kD
18kD
G
si-Ubc9
H
I
Vehicle + vector
LPS + vector
LPS + Ubc9
J
P=0.000
P=0.004
ox-LDL+LPS
Relative SR-A Ⅰ mRNA expression (fold of ox-LDL+vector)
vector
Ubc9
K
P=0.001
P=0.048
L
His-Ubc9
GST-IRF1
GST
Bound
His
Purified protein
M
His-Myd88

Supplemental Figure 6. SUMOylation of IRF1 by Ubc9 Inhibits IRF1 Nuclear Translocation. A, Schematic of the experimental procedure used to identify different IRF1 protein complex in ox-LDL incubated macrophages with or without LPS challenge. **B and C,** Effects of Pam_3CSK_4 **(B)** and Poly (I:C) **(C)** on cholesterol content (***left panel***), SR-AI expression (***middle panel***) and the interaction of IRF1 with SR-AI promoter (***right panel***) in ox-LDL incubated macrophages silenced with si-Myd88 or si-IRF1. **D,** Effects of LPS on the cellular localization of Myd88 in macrophages incubated with ox-LDL, as determined by immunofluorescence assay (***upper panel***) and by Western blot (***lower panel***). **E,** Effects of LPS on the interaction of Myd88 with SR-AI promoter in macrophages incubated with ox-LDL, as determined by ChIP assay. **F,** Ox-LDL incubated macrophages were silenced with scramble siRNA or si-Ubc9, and then the lysates were subjected to immunoprecipitation with the control IgG or an anti-IRF1 antibody followed by immunoblot analysis with antibodies to Myd88 and Sumo1. **G,** The relative mRNA levels of SR-AI in ox-LDL incubated macrophages silenced with scramble siRNA or si-Ubc9. **H,** The interaction of IRF1 with SR-AI promoter in ox-LDL incubated macrophages silenced with scramble siRNA or si-Ubc9, as determined by ChIP assay. **I,** Ox-LDL incubated macrophages were transfected to express Ubc9 or empty vector followed by LPS challenge, and then the lysates were subjected to immunoprecipitation with the control IgG or an anti-IRF1 antibody and analyzed by Western blot with antibodies against Myd88 and Sumo1. **J,** Ox-LDL incubated macrophages were transfected to express Ubc9 or empty vector followed by LPS challenge, and then the relative mRNA levels of SR-AI were examined. **K,** Ox-LDL incubated macrophages were transfected to express Ubc9 or empty vector followed by LPS challenge, then the interaction of IRF1 with SR-AI promoter was determined by ChIP assay. **L and M,** GST pull-down assay was used to analyze the *in vitro* binding of purified GST-fused IRF1 with purified His-tagged Ubc9 **(L)** or Myd88 **(M)**. Data represent the mean ± SEM of three independent experiments. One-way ANOVA with Bonferroni test was used to produce the P values given in figure. * $P < 0.05$. ns, no significance.

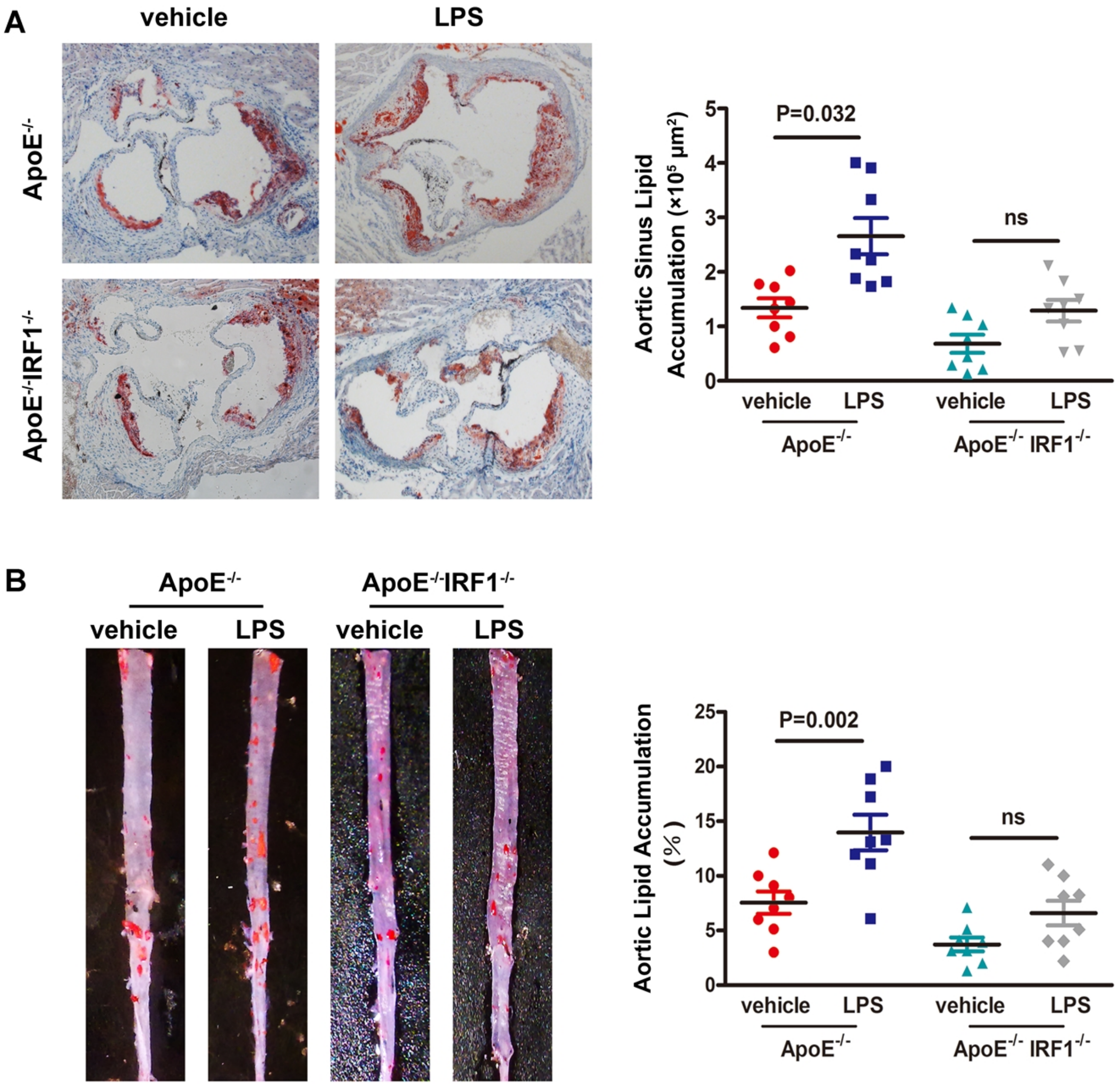

Supplemental Figure 7. IRF1 Contributes to the LPS-induced Aggravation of Atherosclerosis. $ApoE^{-/-}$ and $ApoE^{-/-}IRF1^{-/-}$ mice were fed a western diet for 8 weeks and intraperitoneally injected with either PBS or LPS (5 ng per kg body weight) every 3 days. **A,** Cross-sections of aortic sinus were stained with Oil Red O staining and lipid accumulation was quantified. Scale bar = 200 µm. **B,** Lipid accumulation of thoracoabdominal aorta was evaluated by *en face* staining with Oil Red O, and quantified as percentage of total surface area of aorta. Data are expressed as mean ± SEM (n=8 per group). One-way ANOVA with Tamhanes's T2 test was used to produce the *P* values given in panel **A**. One-way ANOVA with Bonferroni test was used to produce the *P* values given in panel **B**. ns, no significance.
